# Serendipitous sparks: AI information encounter, cognitive flexibility, AI literacy, and university student creativity

**DOI:** 10.3389/fpsyg.2025.1623730

**Published:** 2025-11-26

**Authors:** Xiaoyan Chen, Limin Xiao

**Affiliations:** 1 College of Business Administration, Huaqiao University, Quanzhou, China; 2 School of Business, Sun Yat-sen University, Guangzhou, China

**Keywords:** AI information encounter, cognitive flexibility, AI literacy, creativity, cognitive flexibility theory, human information behavior

## Abstract

As university students increasingly interact with AI, understanding how student-AI interaction behaviors are associated with creativity has gained increasing scholarly attention in recent years. However, previous research has yet to examine the correlation between human information behavior and creativity in AI usage, particularly in relation to information encountered with greater cognitive transformation potential. This study introduces the concept of AI information encounter in the context of student-AI interactions and explores its association with university students’ creativity, including its mechanisms and boundary conditions, through the lens of Cognitive Flexibility Theory. Survey data were collected from 645 university students across different grades, regions, and majors. We complemented PROCESS with CB-SEM and PLS-SEM to triangulate the model, and the convergence across methods supports the model’s stability. The results showed that AIIE positively predicted students’ creativity, with cognitive flexibility serving as a positive mediator. Notably, the mediation of cognitive flexibility was only significant among students with medium to high levels of AI literacy, demonstrating a moderated mediation effect. The findings highlight the relevance of AI information encounters among university students and identify a mediating role linking AIIE to individual creativity, and shed light on practical implications for higher education institutions and teachers to cultivate university student creativity effectively.

## 1 Introduction

Enhancing university student creativity has long been a core mission of higher education, given its growing importance for professional development, personal fulfillment, and lifelong well-being (Moreno and Molero Jurado, 2023; Mariani and Dwivedi, 2024). Within contemporary higher education, generative AI has become a key interface for student information acquisition, and its growing integration into academic life has sparked increasing scholarly interest in how human-AI interaction fosters student creativity (Du Boulay et al., 2023; Jia et al., 2024; Anantrasirichai and Bull, 2022c; Wang S. et al., 2023; Lin and Chen, 2024). As creativity often emerges from how individuals seek, process, and connect information (Demirkol et al., 2025; Sun and Xi, 2024), human information behavior within human-AI interaction becomes especially critical, encompassing both active search and passive encounter (Liu et al., 2022). As generative AI enables increasingly sophisticated and intelligent search experiences (Pham et al., 2024), college students’ interactions with AI are reshaping information seeking from a stable, rule-governed process into one characterized by fluidity, iteration, and uncertainty, often following unpredictable trajectories (Mueller, 2025). In other words, generative AI not only enhances the efficiency of active search but also significantly increases the likelihood of serendipitous information encounters, offering users unexpected yet relevant insights at an unprecedented frequency (Erdelez et al., 2024; Liu et al., 2024).

Information encounter (IE, hereinafter), as a form of passive encounter, refers to the unintentional discovery of unexpected yet valuable information that can facilitate knowledge sharing and spark innovation (Cunha et al., 2015; Thakuria et al., 2024). In the context of human–AI interaction, we extend this concept to AI information encounter (AIIE), which describes individuals’ unplanned encounters with useful information while engaging with AI systems (Erdelez and Makri, 2020; Erdelez et al., 2024). In this study, we specifically focus on generative artificial intelligence (generative AI), such as ChatGPT, Copilot, or similar large language models (LLMs), which provide open-ended, context-sensitive responses based on natural language input. Unlike traditional AI systems such as machine learning-based recommendation engines, which rely on predefined algorithms and structured data to suggest content, generative AI actively synthesizes novel content across diverse domains (He et al., 2025). The interactions it enables are not only information-rich but often surprising and multifaceted (Storey et al., 2025), making them particularly suitable for serendipitous or passive information encounter (Harati and Isfandyari-Moghaddam, 2023). For instance, generative AI could suggest unexpected theoretical perspectives during the analysis of a phenomenon or offer methodological content while providing interdisciplinary cases. Akin to IE’s link with innovation, AIIE could be positively associated with individual creative thinking.

Prior research presents divergent perspectives on AI’s role in fostering individual creativity through information interaction. Some studies argue that AI systems may suppress human autonomy (Fronczek et al., 2023) and original creative impulses (Caporusso, 2023; Anantrasirichai and Bull, 2022; Lin and Chen, 2024), while others suggest that collaborative human-AI partnerships support incremental innovation (Jia et al., 2024), enable decision-making (Suh, 2019), and foster skill improvement (Wambsganss et al., 2022). This discrepancy may result from different types of information behavior, such as active search versus passive encounter, engaging distinct cognitive processes (Agarwal, 2015; Erdelez et al., 2024). When individuals actively seek information, generative AI may replace parts of the creative cognition process, limiting opportunities for original idea generation (Caporusso, 2023; Anantrasirichai and Bull, 2022). Conversely, the passively encountered information might prompt individuals to actively engage in cognitive processing, facilitating cognitive elaboration and creative thinking (Spiro et al., 1988; Spiro, 1987; Makri and Buckley, 2020). Not all modes of information interaction with generative AI are equally beneficial to creativity. Given these divergent findings, this study focuses on the potentially cognitive-transformative information behavior: IE.

Despite increasing interest in AI-assisted learning and creativity, the role of AI-enabled information encounter in enhancing individual creativity remains underexplored. Though existing research has established comprehensive frameworks for facilitating such IE, investigations on how IE predicts individual creativity remain largely scarce, only focusing on collective innovation (Cunha et al., 2015; Thakuria et al., 2024). To fill this gap, the current study thus addresses the following research questions:

*RQ1*: Does AIIE statistically predict creativity?

*RQ2*: What is the psychological process through which this association occurs?

Cognitive flexibility theory (CFT) explains how learners develop cognitive flexibility in complex and ill-structured knowledge domains (Spiro, 1987; Spiro et al., 1988). Given the unstructured and diverse nature of AI-generated context, CFT provides a valuable framework for understanding the cognitive processes that AIIE elicits. AI-generated information introduces variety and unpredictability, disrupting fixed models of information seeking and engaging students in a more dynamic, iterative cognitive process (Mueller, 2025). Unlike regular and fixed knowledge units, unexpected and multifaceted information could transform an individual’s knowledge representation (Spiro et al., 1988), as novel inputs are assimilated into existing schemas, fostering more flexible and dynamic knowledge representations (Spiro et al., 1988; Spiro, 1987). By making the information-seeking process less linear and more reflexive, generative AI challenges the notion of a singular, authoritative research path, compelling students to actively navigate the evolving landscape of information (Mueller, 2025). Enhanced cognitive flexibility places students on a critical pathway to creative thinking (Kwon and Kim, 2025; Chen et al., 2019).

Notwithstanding the creativity-enhancing potential of AIIE, its actualization remains conditional upon individuals’ capacity to discern informational salience. AI literacy reflects students’ ability to identify, use, evaluate, and ethically govern AI output (Kandlhofer et al., 2016; Wang B. et al., 2023). Whether individuals can extract useful information units determines subsequent usage of this knowledge. Though AI-generated content is more synthesizing and diverse, AI-fabricated misinformation, counterfactuals, untruths, and misinformation overflows more frequently than other media today (Garimella and Chauchard, 2024; Erdelez et al., 2024; Liu et al., 2022; Mueller, 2025). In this sense, the competence to discern valuable information emerges as an essential “filter” in developing creativity. Crucially, beyond technical competence, AI literacy must also address the psychological tensions students experience when balancing convenience with integrity, as generative AI use can trigger cognitive dissonance in academic contexts (Seran et al., 2025). Thus, the boundary condition also constitutes our research purpose:

*RQ3*: What’s the boundary condition of the association between AI information encounters and creativity?

To address the above research questions, this study aims to explore the effect of AIIE and creativity as well as the psychological mechanism, and investigate AIIE’s role among students with different AI literacy levels. Grounded in Cognitive Flexibility Theory, we study a conditional process linking AIIE to creativity via cognitive flexibility. AI literacy is hypothesized to condition this process by moderating both the path between AIIE and cognitive flexibility and the direct association between AIIE and creativity. Accordingly, the indirect association between AIIE and creativity through cognitive flexibility could be moderated by AI literacy. We validated it through a survey of 645 students across different genders, grades, and majors, and adopted CB-SEM, Process model, and PLS-SEM to examine the results. This research provides significant contributions. We captured a novel human information behavior, AI information encounter, and advanced the theoretical framework in understanding how human-AI interaction stimulates individual creativity, unveiled the psychological mechanism, and pioneered the boundary of Cognitive Flexibility Theory. From a practical perspective, our findings shed light on how educational institutions can effectively leverage AI-related information facilitators and cognitive cultivation to shape university student creativity.

## 2 Literature review and theoretical foundation

### 2.1 AI information encounter

Information encounter (IE) was first introduced to denote encountering valuable information without the deliberate search efforts of the individual (Erdelez, 1995). Individuals may encounter useful and interesting information while seeking or browsing for other unrelated information (Erdelez and Makri, 2020). As a form of human information behavior, IE differs from purposeful and intentional information searches; instead, it represents passive encounters characterized by minimal or no intentionality, involvement, or expectation in obtaining information (Garimella and Chauchard, 2024).

Based on the definition of IE (Erdelez and Makri, 2020), AI information encounter (AIIE) in this study was defined as “finding interesting, useful, or potentially useful information when one is engaging with any other theme in interaction with AI, or not intentionally looking for information at all.” AI enables unprecedentedly frequent IE by processing large real-time datasets and providing multi-faceted information. Unlike traditional media, AI systems provide continuous, context-sensitive responses that are associated with both higher frequency and relevance of information flow (Khan et al., 2023). Their fluidity and unpredictability create more opportunities for unexpected insights and routine-breaking thinking (Mueller, 2025). In higher education, such interactions can foster information encounter in learning and academic life, such as unanticipated theoretical comprehension, research development, and methodological innovation.

We capture the connotation of AIIE based on traditional understandings of information encounter (IE). To further clarify the conceptual grounding of AIIE, we elaborate on its alignment from 3 aspects: environment, feature, and information processing. First, both forms of encounter occur in *rich and complex* information environments. Traditional IE often takes place in libraries, search engines, or online forums curated by human contributors, while AIIE occurs in interactions with generative AI systems that produce diverse, unstructured, and context-sensitive outputs. Both contexts expose individuals to a wide array of information, making the occurrence of unexpected insights possible.

Second, the *unintentionality* of the information encountered in both IE and AIIE fundamentally supports their classification as passive information behaviors. It is important to clarify that information encounter typically occurs embedded within broader information-seeking or browsing activities. Individuals do not experience serendipitous discovery at every moment of interaction with a search engine or generative AI. Instead, “valuable and unexpected information encountering” often emerges while users are pursuing other goals or browsing for other unrelated information (Erdelez and Makri, 2020). In other words, the presence of an initial goal does not preclude the occurrence of IE; rather, the defining feature is whether serendipitous content arises during the process. Similarly, generative AI may introduce unexpected insights (Storey et al., 2025), such as surprising theoretical perspectives, interdisciplinary examples, or alternative methods while addressing a specific query. This unpredictability reflects the essence of IE, reinforcing AIIE as a form of passive encounter.

Third, both IE and AIIE involve shared *information processing phases*, modeled through four sequential phases: noticing, stopping, checking, and capturing (Erdelez, 1995, 2004; Kim et al., 2023). AIIE, like traditional IE, involves individuals’ cognitive responses to unexpected informational stimuli, specifically, how they shift attention from their original goals to evaluate and engage with newly surfaced content. Building upon Kim et al.’s (2023) theoretical construct, this study also operationalizes AIIE as a four-dimensional process. First, during the noticing phase, users exhibit initial recognition of serendipitous information generated by AI systems. In the stopping phase, they temporarily suspend their original task to further attend to the new content. The checking phase involves deeper exploration, often verifying or cross-referencing the emergent information. Finally, in the capturing phase, individuals integrate and apply this information to ongoing work or share it with others (As shown in Table 1). Importantly, this model has been consistently applied across both analog and digital contexts (Agarwal, 2015; Erdelez et al., 2024), paving the way for a legitimate extension of the IE framework in generative AI interactions. In this light, AIIE preserves the theoretical core of traditional IE while expanding its relevance to AI-mediated environments. Based on Kim et al.’s (2023) four-phase information encounter model, we identified common scenarios in which university students encounter unexpected information during AI interactions, and exemplified how students engage in noticing, stopping, checking, and capturing phases within each scenario. Table 1 presents these contexts, including theory extension, interdisciplinary cases, code debugging, language learning, and career exploration, along with corresponding examples for four-phase behaviors.

**Table 1:** Representative examples of AI information encounter across four dimensions.

Scenario	Dimensions of AI information encounter			
	Noticing	Stopping	Checking	Capturing
Theoretical extensions in paper writing	When students inquire about a specific theory using conversational AI, they notice new theories mentioned by the AI.	Suspend the original plan for writing the paper.	Request AI to explain in detail the relationship between these two theories and cross-verify with the literature.	Integrate the paper and share it with the academic community.
Interdisciplinary case recommendations when studying a specific methodology	Use an AI search engine to look up a specific method and notice the interdisciplinary case studies recommended by AI.	Stop the methodology study and click to view the case summary generated by AI.	Trace back through AI citations to obtain source information and gain a deeper understanding.	Integrate interdisciplinary case studies into the course presentation PPT and discuss them with the teacher and classmates.
Code debugging	When debugging Python code using an AI programming assistant, the system prompts to try a new algorithm.	Stop debugging the code.	Compare the current new method with the expected method and test by sandbox simulation.	Write the new method in the appendix of the experiment report.
Language learning breakthrough	While practicing English speaking with the AI assistant, the AI assistant suggested changing the sentence structure.	Interrupt the preset dialog flow and ask the AI to display more examples of different contexts.	Use AI to simulate dialog variations in different contexts, such as a café, classroom, or academic conference.	Organize these scenarios and create a flashcard activity in the language improvement group.
Career development exploration	When students inquire about the requirements for a specific job, recommend similar emerging career paths.	Postpone the planned resume revision and search for new career information.	Retrieve the job demand trends of the past three years through the AI industry database.	Send the career development roadmap created by AI to friends and family for discussion.

Each row corresponds to the original 5 scenarios. Horizontal reading restores the complete case chain, while vertical reading reflects the common dimensional characteristics.

In previous studies on IE, the consequential outcomes have been inadequately explored, particularly concerning individual-level creativity. Existing research on IE alongside its similar concept, information serendipity (Liu et al., 2024; Sun et al., 2022), has accumulated well-established exploration of the antecedents, which could be classified into 3 categories: (a) user-related factors, e.g., readiness, curiosity, and motivation (Björneborn, 2017; Busch, 2024; Lievens et al., 2022); (b) individual factors, e.g., information literacy (Stewart and Basic, 2014) and propensity (Kim et al., 2023); (c) task-related dimensions, encompassing task type (Erdelez, 2004) and characteristics (Jiang et al., 2022). However, the outcomes of IE have been under-investigated, especially in terms of individual creativity. Though IE has proven to be associated with higher knowledge sharing and the formation of innovative synergies within the organization (Cunha et al., 2015; Thakuria et al., 2024), the association between IE and creativity at the individual level has been overlooked. In AI–human interaction contexts, how AIIE relates to individual creativity remains to be explored.

### 2.2 AI literacy

AI literacy (AIL, hereinafter) was defined as “the ability to properly identify, use, and evaluate AI-related products under the premise of ethical standards” (Wang B. et al., 2023). AIL encompasses 4 elemental dimensions: (1) identification, (2) use and application, (3) evaluation, and (4) ethics (Wang B. et al., 2023). Existing studies have mainly focused on the measurement of AI literacy and the factors in cultivating AIL, while the outcomes of AI literacy have remained under-investigated, especially in the education domain (Carolus et al., 2023; Ng et al., 2024; Casal-Otero et al., 2023). Different from information literacy (Stewart and Basic, 2014) in general human information behavior, AIL aims to address emergent challenges within human-AI interaction contexts. As methods to handle the rapidly expanding volume of AI-generated information remain underdeveloped, AIL has emerged as a critical competency in modern education (Ng et al., 2024). Therefore, when students are exposed to unexpected and potentially confusing information from AI, AIL plays a key role in enabling them to identify, evaluate, and retrieve useful information.

### 2.3 Cognitive flexibility

Cognitive flexibility (CF, hereinafter) refers to a person’s (a) awareness that options and alternatives exist in any given situation, (b) willingness to be flexible and adapt to the situation, and (c) self-efficacy or belief that one can be flexible (Martin and Rubin, 1995). Previous research primarily focused on the relationship between CF and individuals’ lives and learning, revealing significant benefits ranging from academic achievement to improved resilience, handling of negative emotions, and well-being (Diamond and Lee, 2011; Dajani and Uddin, 2015). In terms of antecedents, previous works emphasized personal traits and experience (Chung et al., 2012; Jacques and Zelazo, 2013) but overlooked the human information behavior stimuli, such as information encounter. The current study endeavors to bridge the existing theoretical framework and the unexplored antecedents.

More importantly, research on the relationship between cognitive flexibility (CF) and creativity has remained a central focus, as the two are closely linked through individuals’ capacity for perspective-shifting and divergent thinking (Kwon and Kim, 2025; Chen et al., 2019; Dietrich and Kanso, 2010; Hohl and Dolcos, 2024). However, when and how CF is linked to higher creativity remains underexplored. While AI Literacy refers to a domain-specific technological competence, CF represents a broader psychological capacity. When individuals encounter external stimuli, their ability to handle the information source in context (e.g., an AI-generated environment) shapes how new informational units are integrated, thereby relating to cognitive flexibility. In our study, the dynamic interaction between AIL (context-specific competence) and cognitive disposition may be positively associated with higher creativity.

### 2.4 Individual creativity

Fostering creativity is often viewed as a central goal of the educational system in many countries, as it is an important source of science and technology innovation in modern societies (Castillo-Vergara et al., 2018). Innovation and creativity share some common characteristics, but they also hold significant distinctions. The former refers to the action of new idea generation, whereas the latter refers to the conversion of a new idea into an end output (Perry-Smith and Mannucci, 2017). Following the traditional conceptualization, the current study defines creativity as the capacity to produce novel or original outputs that are also useful or appropriate (Amabile, 2019; Plucker et al., 2004; Runco and Jaeger, 2012; Harvey and Berry, 2023). The connotation of creativity was also considered in line with the scope of university students.

To contextualize creativity within the university student population, this study focuses on creative behaviors that typically occur in students’ daily life and academic experiences, such as discussion, writing, course assignments, critical thinking, research exploration, etc. These forms of creativity are neither professional-level accomplishments nor breakthrough innovations, but rather part of students’ ongoing intellectual development. In line with this perspective, we refer to the 4C model of creativity proposed by Kaufman and Beghetto (2009), which categorizes creativity into four levels: mini-c (personal insight and meaning-making), little-c (everyday problem-solving and expression), Pro-c (professional-level creativity), and Big-C (eminent, transformative creativity).

As creativity denotes the use of knowledge and the ability to generate novel and useful ideas (Gong et al., 2009), it is crucial to consider the role of information as a foundational input in the creative process. Innovation is rarely the product of isolated insight; rather, it is typically built upon the reconfiguration of existing informational and knowledge-based resources (Wang and Nickerson, 2017). Prior studies in organizational and cognitive science have emphasized that new ideas often emerge through the reinterpretation, recombination, and transformation of past knowledge (Benedek et al., 2023). In this sense, information functions not merely as raw material but as a cognitive resource that interacts with pre-existing schemas to facilitate conceptual restructuring. This perspective is particularly relevant for university students, whose creative output is predominantly rooted in knowledge-intensive tasks, such as academic writing, theoretical reflection, or research design.

Previous research has explored various information-related drivers of creativity, including domain-specific information from active search (Palani et al., 2021), accessibility of domain knowledge (Rietzschel et al., 2007), memory search (Benedek et al., 2023), information richness (Wang and Nickerson, 2017), and interdisciplinary input (Perry-Smith and Mannucci, 2017). However, these studies primarily focus on intentional, structured, and goal-directed information acquisition, overlooking the creative potential of passively encountered information.

Serendipitous or unexpected information, particularly when unstructured or outside the user’s original scope, can serve as a powerful cognitive stimulus. Such inputs can challenge existing thought patterns, promote integrative thinking, and spark novel connections (Kleinmintz et al., 2019; Benedek et al., 2023). As Palani et al. (2021) and Wu et al. (2014) emphasize, creativity depends not only on access to information but on how individuals engage with it cognitively. Understanding how non-intentional information inputs contribute to creative thinking thus offers critical insight into the micro-foundations of student innovation.

### 2.5 Cognitive flexibility theory

Cognitive flexibility theory (CFT) is a learning and teaching theory that explains how learners acquire more flexible cognitive structures and apply advanced knowledge in complex and ill-structured knowledge domains (Spiro, 1987; Spiro et al., 1988). The ill-structured knowledge represents a complex and multifaceted information landscape for the learner. In the irregular and hypertext domain, general principles would become increasingly indeterminate in capturing enough of the dynamics of concepts and perspectives in an irregular and unfamiliar information landscape.

As ill-structuredness increases, fixed, pre-compiled knowledge structures would be supplanted by more dynamic, combinable cognitive units (Spiro, 1987). Along schema segments in relation, newly encountered information is integrated with existing knowledge across multiple conceptual dimensions. In this way, individuals form adaptive cognitive frameworks in response to different situations, or “situational schema assemblies” (Spiro et al., 1988). One could have many alternative paths to get from one node of the overall knowledge base to any other node. Thus, cognitive flexibility is fostered where one can represent knowledge from various perspectives, and tailor knowledge ensemble to specific situations or problem-solving contexts (Spiro et al., 1988; Spiro et al., 2013).

The aim of exploring how AIIE (involving bewildering information) relates to individual creativity is in line with CFT, which clarified the cognitive process of how ill-structured knowledge domains are positively associated with individual flexible schema and advanced knowledge transfer (Dang et al., 2021). Thus, CFT serves as an appropriate lens in elucidating the cognitive process when students experience AIIE, including the boundary and mechanism of how AIIE effectively affects creativity.

## 3 Hypothesis establishment

### 3.1 AI information encounter and university student creativity

Creativity reflects the use of knowledge and the ability to generate novel and useful ideas (Gong et al., 2009). More specifically, creative performance involves three core cognitive processes: cognitive restructuring, novelty evaluation, and contextual fit (Harvey and Berry, 2023; Kleinmintz et al., 2019). In this context, the quality and nature of information input are associated with higher creativity (Wu et al., 2014; Benedek et al., 2023; Palani et al., 2021). Serendipitous resources, as a broader form of information serendipity, have been shown to facilitate organizational innovation by disrupting established trajectories, prompting schema revision, and stimulating new dialogs between strategic plans and emerging possibilities. Serendipity reflects openness, refreshes organizational memories and routines, countering directional crystallization, challenges dominant logics, and encourages organizations to reinterpret unexpected resources as opportunities for change (Cunha et al., 2015; Kim and Zhong, 2017).

At the individual level, AIIE, as a form of information serendipity, can similarly serve as a catalyst for creativity in its three cognitive facets. First, the unstructured and diverse content generated by generative AI may disrupt pre-existing mental schemas, thereby prompting *cognitive restructuring*, a fundamental precursor to creative thinking (Spiro et al., 1988; Dheer and Lenartowicz, 2019). Just as organizations adapt and revise routines in response to serendipitous events, individuals may reinterpret AI-generated information to rebuild and expand their own knowledge frameworks. Second, the serendipitous perspectives or interdisciplinary insights provided by AI can stimulate *novelty evaluation*, requiring users to assess the originality, coherence, and relevance of unfamiliar content (Kleinmintz et al., 2019). Much like how firms challenge existing strategies when exposed to novel inputs, individuals may experience a cognitive reevaluation of ideas that fall outside their expected information scope (Cunha et al., 2015; Kim and Zhong, 2017).

Third, the adaptive nature of AI-generated responses enables users to explore *contextual fit*, experimenting with how newly surfaced ideas can be applied in academic, technical, or personal domains (Amabile, 2019; Harvey and Berry, 2023). In this sense, AIIE does not merely deliver informational input; it triggers reflective thinking, schema adaptation, and creative recombination. For example, when interacting with a generative AI assistant, a student may unexpectedly receive theoretical extensions while inquiring about a familiar framework. Through further dialog and iterative prompts, the student collaborates with the AI to explore whether and how the new theory fits in the specific academic or practical context. Likewise, when discussing job prospects, students may be introduced to alternative career paths they had not previously considered. By examining labor market data or role comparisons synthesized by the AI, they can assess the feasibility and relevance of these new directions.

As Foster and Ford (2003) noted, such moments “can open your eyes to a whole new set of views,” thereby stimulating divergent thinking and creative ideation. In other words, AIIE can provide original possibilities for novel outputs (Kleinmintz et al., 2019; Harvey and Berry, 2023). Thus, we propose the following hypothesis:

*H1*: AI Information Encounter (AIIE) is positively associated with university student creativity.

### 3.2 The mediation effect of cognitive flexibility between IE and creativity

According to CFT, students obtain cognitive flexibility from processing ill-structured and hypertext information (Spiro et al., 1988). Similarly, the novel information in AIIE for students is unstructured and multifaceted, as AI-generated or AI-recommended content is beyond explanation and more diversified than regular learning materials. In other words, AIIE moves beyond intact, rigid, precompiled knowledge structures. For students confronted with novel and unexpected knowledge, general principles will not capture enough of the dynamics of information units. In gaining cognitive flexibility, the “storage of fixed knowledge is devalued in favor of the mobilization of potential knowledge” (Schank, 1982; Spiro et al., 1988). Pre-packaged schema would be transformed into mobilizable segments, and integrated with new conceptual elements as a more dynamic pattern of combination. In this way, students gain a cognitively flexible mindset to set different yet interconnected assemblies of knowledge to adaptively fit the situation at hand.

Cognitive flexibility shows the ability to break old cognitive patterns, overcome functional fixedness, and thus, make novel (creative) associations between concepts (Guilford, 1967). The capacity to switch between different cognitive styles enables individuals to make decisions in environments characterized by high complexity and uncertainty (Dheer and Lenartowicz, 2019), which implies the potential for divergent thinking and innovative perspectives. Creativity, as an indicator of useful and novel output, requires flexibility in concept networking and divergent cognitive thinking (Baas et al., 2008), which aligns with the features of cognitive flexibility. Cognitive flexibility helps individuals to identify choices and alternatives available in any situation and adapt to the present with divergent cognitive routes (Martin and Rubin, 1995). Hence, cognitive flexibility serves as a premise of creativity. As cognitive flexibility bridges IE and creativity, we propose the following hypothesis

*H2*: Cognitive flexibility accounts for an indirect association between AI information encounter (AIIE) and creativity.

### 3.3 The moderation role of AI literacy

The key role of AIIE in fostering individual creativity manifests in fostering cognitive restructuring, novelty evaluation, and the contextual fit of innovation outcomes (Harvey and Berry, 2023; Kleinmintz et al., 2019). To ensure the conditions for creativity, individuals must possess the ability to actively discern and evaluate the elements of information that effectively expand cognitive boundaries and make adaptations that fit the context at hand. In student-AI interaction, AI literacy reflects students’ ability to identify, evaluate, and retrieve information during AI-mediated interactions with unanticipated information (Wang B. et al., 2023). We argue that AI literacy amplifies the efficacy with which students utilize useful parts from unexpected information and boosts individual creativity.

First, AI literacy facilitates individuals’ better utilization of information resources that aid cognitive restructuring. Complexity and irregularity can challenge the routinization of knowledge, but this requires a reflective dialog and collaborative process (Mueller, 2025). Novel concepts and existing knowledge often deviate from each other, leading to an acceptance cost. The “motivation” for cognitive development stems from bridging the gap between existing schema and the understanding of the world, thus reconstructing new perspectives on unfamiliar yet necessary things (Bjorklund, 2018). Only when individuals recognize unfamiliar information as necessary and valuable will they be motivated to engage in accommodation and restructure their interpretations of existing knowledge. AI literacy reflects an individual’s ability to better identify effective information (Wang B. et al., 2023; Ng et al., 2024), thereby promoting cognitive expansion and restructuring.

Second, AI literacy better ensures individuals’ novelty evaluation. Given that AI information is often biased or misleading (Garimella and Chauchard, 2024; Erdelez et al., 2024; Liu et al., 2022), assessing the authenticity and innovation potential of information is crucial for creativity. Third, AI literacy satisfies the premise of contextual fit. Individuals with high AI literacy are adept at retrieving appropriate operational resources, enabling them to utilize AI-generated cross-disciplinary cases, novel methodologies, and unexpected career recommendations to co-create suitable solutions to current problems. In contrast, students with low AI literacy may struggle to identify and evaluate the valuable parts of the multifaceted information provided by AI, potentially being misled by false information. This makes cognitive restructuring difficult, and they are unlikely to extract resources that are contextually appropriate, thereby underperforming in terms of innovation. Hence, we propose the following hypothesis:

*H3*: AI literacy moderates the association between AIIE and creativity, such that the effect is stronger at a high level of AI literacy than at a low level.

### 3.4 The moderated mediation model

Cognitive flexibility is dependent upon having a diversified repertoire of ways of thinking about a conceptual topic (Spiro et al., 1988). From a CFT perspective, hypertext and ill-structured information help learners move beyond fixed schemas; in such domains, broad rules rarely capture case dynamics, so flexibility grows through responding to varied, novel instances. We posit that AI literacy (AIL) acts as a filter that turns ill-structured input into usable building blocks for CF. When learners can evaluate and extract meaningful content, discrete knowledge units (both new and existing) become modular components for constructing more dynamic, networked representations (Spiro et al., 1988). Novelty alone is insufficient; flexibility emerges when information is critically appraised and integrated.

When confronted with abundant, ill-structured information, students with higher AIL can discern, select, and retrieve connectable elements aligned with their existing representations (Steiner and Stoecklin, 1997). By evaluating the innovative potential of these elements and integrating them, they reconfigure schemas into more networked, flexible structures that support novel, context-sensitive ideas. Conversely, students with low AIL struggle to filter and extract what is useful; the influx is experienced as confusing or irrelevant noise, closing off adaptive routes (Thakuria et al., 2024; Heinström et al., 2020). Difficulty linking alternatives hampers the development of cognitive flexibility and, in turn, limits creative output.

The proactivity in internalizing knowledge is key to expanding cognitive schemas. Cognitive flexibility develops when learners actively abstract knowledge through learner-driven processes (e.g., case analysis, example-based reasoning) rather than passively receiving fixed frameworks (Steiner and Stoecklin, 1997). Within this, the ethical dimension of AIL is pivotal yet often overlooked: it orients users toward reflective appraisal of AI outputs and preserves autonomy in how information is selected and used (Bankins and Formosa, 2023). Without ethical AIL, generative AI’s powerful affordances can dilute human control and accountability, weakening motivation to integrate unexpected information into existing schemas and hampering CF (Bankins and Formosa, 2023; Ardichvili, 2022). Without checking relevance, accuracy, or bias, unreflective reliance on AI undermines cognitive agency and constrains creative output. By contrast, high ethical AIL fosters a sense of responsibility and deliberate engagement, encouraging learners to mobilize cognitive resources and broaden knowledge structures for further creativity.

*H4*: AI literacy moderates the association between AIIE and creativity via cognitive flexibility, such that the indirect association is stronger at a high level of AI literacy than at a low level.

**Figure 1:**
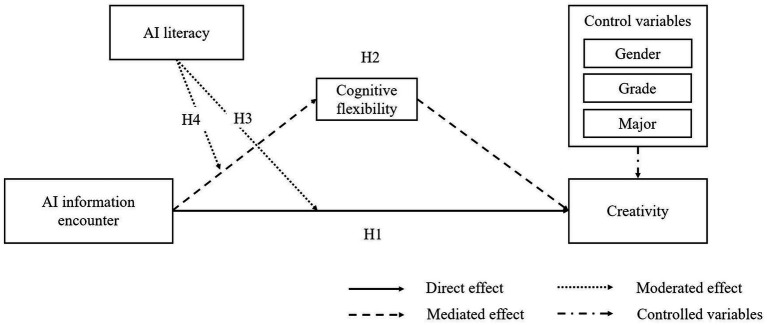
The conceptual framework of this research.

Figure 1 illustrates the conceptual framework of the study. Grounded in cognitive flexibility theory, AIIE is posited to be positively related to creativity via CF. Ill-structured inputs expand and reconfigure knowledge structures, enabling novelty. AIL might function as a filter that shapes how learners evaluate and integrate AIIE inputs, thereby moderating both the AIIE–CF pathway and the overall AIIE–creativity link.

## 4 Methods

### 4.1 Data collection

A cross-sectional survey design was employed to collect data, which has been shown to be effective for achieving high external validity in information behavior research (Awan et al., 2020; Liu et al., 2022). Given that prior scholars have emphasized the importance of examining IE across diverse cultural and educational contexts, a large and demographically varied sample was necessary to ensure generalizability.

Data were collected from Credamo, a leading data collection platform in China that serves over 3,000 academic institutions and operates similarly to Qualtrics and MTurk. Its database includes 1.5 million respondents across diverse demographics.^1^ Credamo ensures high-quality and effective data collection through check questions, target sample positioning, IP monitoring, and captchas, rewarding participants only upon passing, which has been widely used by scholars (Tang et al., 2023). We conducted the survey in two phases. Since AIIE is a new construct captured in this study, we first conducted a pre-survey on Credamo between August 10 and August 14 with a total of 400 university participants. This pre-survey aimed to develop a reliable and validated measurement scale for AIIE (see Appendix A).

The second phase of data collection took place between August 24 and August 30, 2024, with a total of 700 university participants recruited. This phase was intended to validate our theoretical model and hypotheses, which is the primary focus of this research. Each participant was only allowed to take the survey once. The first question assessed whether participants had used AI for information seeking. Participants who met the inclusion criteria were asked to fill in an online survey. Attention-check questions in the questionnaire items to identify unreliable data. 5 yuan was paid at the end of the survey as feedback for participants to ensure that they answered carefully. Furthermore, the judgmental sampling method was adopted in this study, assisting in selecting valid samples and minimizing non-response bias (Rowley, 2014). Individuals who did not meet the criteria above were excluded. Finally, a total of 645 questionnaires (women = 70.23%; men = 29.77%) were collected, with a survey questionnaire validity rate of 92.14%. 26.20% freshman and sophomore, 28.99% junior and senior, 39.07% postgraduate, and 5.74% doctoral. Regarding disciplinary mix: 46.82% STEM and engineering, 24.03% business and management, 17.36% humanities and social sciences, and 11.78% arts and sports. Table 2 shows the descriptive statistics of respondents.

**Table 2:** Socio-demographic profiles of respondents (*n* = 645).

Characteristics	Category	Frequency	Percentage (%)
Gender	Male	192	29.77
	Female	453	70.23
Grade	Freshman and sophomore	169	26.20
	Junior and senior	187	28.99
	Postgraduate	252	39.07
	Doctoral student	37	5.74
Major domain	STEM and engineering	302	46.82
	Business and management	155	24.03
	Humanities and Social Sciences	112	17.36
	Arts and sports	76	11.78
Total		645	100

### 4.2 Measurements

The items for each construct were adapted from existing, well-developed measurements. Two educational experts and two behavioral experts have examined the content validity of whether the items reflect the connotations of the construct. To translate the original measurement items in English into Chinese, the participants’ mother language, forward-back translation was employed to ensure the accuracy (Beaton et al., 2000). In addition, each instrument was bilingual, double-blind translated, and pre-tested by three experts from Chinese universities. Participants were asked to rate the items according to their own experience. The survey questionnaire was rated on a 5-point Likert scale (where 1 = strongly disagree and 5 = strongly agree).

AI Information Encounter (AIIE) refers to the incidental discovery of interesting, useful, or potentially useful information during interactions with generative AI, particularly when the individual is engaged with unrelated themes or not actively seeking information. Following a rigorous scale development process, we have adopted a pilot survey methodology to develop and validate a reliable measurement scale for AI information encounter (AIIE), which was adapted from the scale of Information Encounter by Wise et al. (2012) and Kim et al. (2023). The EFA and CFA results show AIIE was a construct with four sub-factors pertaining to the same second-order factor of the four-phase model proposed by Kim et al. (2023). The scale development process and results were reported in detail in Appendix A. A 12-item pool spanning four dimensions (noticing, stopping, checking, capturing) was adapted from prior work and refined via expert and lay review. We applied conservative EFA retention thresholds: primary loading > 0.60 and cross-loading < 0.30; all 12 items were retained. A second sample supported a second-order CFA with good fit (*χ*^2^/df = 2.16; CFI = 0.982; RMSEA = 0.016), confirming AIIE as a higher-order construct. Reliability and validity were satisfactory (*α* = 0.801–0.890; CR > 0.70; AVE > 0.50).

The final AIIE scale comprises 12 items across four dimensions: noticing, stopping, checking, and capturing. The four AIIE subscales comprise 4, 2, 3, and 3 items, respectively. Each illustrated with an example item: noticing (“When using generative AI, I notice content that is unrelated to my query goal”); stopping (“While using generative AI, I pause to examine information that is unrelated to my original query”); checking (“When I encounter interesting, unexpected information, I ask the AI for more related content to probe it further”); and capturing (“I share interesting AI-generated content with my family, friends, or colleagues, even if it is not what I originally requested”).

Cognitive Flexibility refers to an individual’s awareness of alternative options, willingness to adapt to changing situations, and confidence in their ability to be flexible (Martin and Rubin, 1995). Cognitive flexibility was measured and adapted from the Cognitive Flexibility Scale (CFS) by Martin and Rubin (1995) and the revised version of Qi et al. (2013), which consists of 12 items in three dimensions: awareness, willingness, and self-efficacy in being flexible. An example item is: “I can express an idea in many different ways.”

AI Literacy is operationally defined as the ability to appropriately identify, use, and evaluate AI-related products within an ethical framework (Wang B. et al., 2023). AI literacy was measured using the Wang B. et al. (2023) scale, the first scientifically rigorous psychometric scale for AI literacy, which consists of 12 items and includes four dimensions: identification, use and application, evaluation, and ethics. An example item is: “I can proficiently use AI applications or products to help me complete daily tasks”.

Creativity is operationally defined as the ability to generate novel and useful ideas through the application of knowledge (Gong et al., 2009). Following Tan et al. (2021) and Zhang et al. (2023), the creativity measurement was adapted from the five-dimensional Kaufman Domains of Creativity Scale, which is more compatible with university students in an Eastern cultural context (Kaufman, 2012; Sen and Yörük, 2023). We retained four dimensions: everyday creativity (“Maintaining a good balance between my work and my personal life”), scholarly creativity (“Responding to an issue in a context-appropriate way”), scientific creativity (“Figuring out how a machine works”), and artistic creativity (“Appreciating a beautiful painting”). In consultation with one education expert and three doctoral students, we excluded the “performance creativity” dimension, as it primarily reflects domain-specific musical abilities (e.g., “Playing music in public”) and lacks content validity for representing general, non-specialized creative ability. Since musical performance is part of the broader artistic domain, our retained artistic creativity dimension continues to reflect attention to artistic expression. The inclusion of these four dimensions ensures that the measurement remains inclusive and balanced.

### 4.3 Data analysis and results

We employed three complementary frameworks to ensure measurement rigor and robust inference. CB-SEM tests reflective measurement and overall fit; PROCESS estimates the moderated mediation with bootstrap CIs for clear conditional indirect effects and simple slopes; PLS-SEM offers a prediction-oriented, distribution-robust check with out-of-sample assessment. Convergent results across covariance-, regression-, and variance-based approaches would strengthen coherence and conceptually triangulate the findings, suggesting the associations are unlikely to result from a single estimation paradigm.

#### 4.3.1 Common method bias

To mitigate potential common method bias (CMB) arising from administering many items within a single session, we adopted several procedural remedies: (a) block-randomized presentation of measures and (b) psychological separation of predictors and outcomes by inserting demographic questions and an attention check between them (Podsakoff et al., 2003). We also collected responses continuously on the Credamo platform from August 24 to August 30, 2024, at varied times of day to increase temporal heterogeneity and strengthen sample robustness.

To assess the multicollinearity, the variance inflation factor (VIF) values for all independent variables were tested. VIF values ranged from 1.048 to 1.304 (see Table 3), well below the critical value of 3.33, demonstrating virtually no multicollinearity (Hair et al., 2021). To assess common method bias (CMB), Harman’s single-factor test and the full collinearity test were employed. Harman’s single-factor test revealed that the variance explained by the first factor was 27.11%, well below the 40% threshold, indicating no significant CMB issue (Fuller et al., 2016). Both tests confirmed the absence of common method bias (CMB) in this study.

**Table 3:** Confirmatory factor analysis for the constructs of the measurement model.

Latent variable	Indicators	Standardized factor loading	Cronbach’s *α*	CR	AVE	VIF
AI information encounter	Noticing	0.864***	0.918	0.917	0.737	1.048
	Stopping	0.873***				
	Checking	0.853***				
	Capturing	0.852***				
Cognitive flexibility	Awareness	0.852***	0.869	0.870	0.690	1.304
	Willingness	0.809***				
	Self-efficacy	0.830***				
AI literacy	Identification	0.807***	0.868	0.852	0.658	1.273
	Use and application	0.806***				
	Evaluation	0.821***				
	Ethics	0.843***				
Creativity	Scholarly	0.792***	0.903	0.903	0.701	–
	Scientific	0.884***				
	Artistic	0.864***				
	Everyday	0.873***				

We implemented a latent method factor (LMF) test in AMOS 24.0 by adding an orthogonal method factor (variance fixed to 1) that loaded on all indicators to the baseline measurement model. Relative to the baseline, overall fit improved only marginally (ΔCFI = 0.002, ΔTLI = 0.002, ΔRMSEA = −0.003; CFI from 0.982 to 0.984, TLI from 0.978 to 0.980, RMSEA from 0.041 to 0.039). These results suggest that common method variance is unlikely to drive the findings (Podsakoff et al., 2003).

#### 4.3.2 Measurement model test

We first conducted an exploratory factor analysis (EFA) to find the essential structure of multivariate observed variables. EFA reduces complex variable relationships into key factors. A commonality above 0.4 indicates good factor representation; KMO values above 0.5 suggest suitability for factor analysis, with values above 0.8 indicating strong suitability (Howard, 2016). Table 4 shows a KMO of 0.889 and all commonalities above 0.4, confirming the indicators are well-suited for capturing target variables.

**Table 4:** Exploratory factor analysis.

Item	Factor 1	Factor 2	Factor 3	Factor 4	Commonality
AIIE1	**0.878**	0.052	0.051	0.164	0.804
AIIE2	**0.890**	0.066	0.031	0.144	0.818
AIIE3	**0.876**	0.069	0.018	0.168	0.800
AIIE4	**0.866**	0.075	0.054	0.174	0.788
CF1	0.077	**0.857**	0.174	0.216	0.818
CF2	0.079	**0.817**	0.217	0.221	0.769
CF3	0.080	**0.833**	0.228	0.199	0.791
AIL1	−0.002	0.158	**0.826**	0.172	0.737
AIL2	−0.001	0.181	**0.842**	0.121	0.755
AIL3	0.132	0.200	**0.783**	0.263	0.740
AIL4	0.041	0.128	**0.789**	0.150	0.663
CRE1	0.141	0.202	0.206	**0.794**	0.733
CRE2	0.192	0.205	0.182	**0.842**	0.822
CRE3	0.251	0.196	0.178	**0.796**	0.767
CRE4	0.170	0.163	0.198	**0.830**	0.784
Eigen root value (before rotation)	6.079	1.271	1.446	2.794	
Explanation of variance % (before rotation)	40.527	8.474	9.638	18.624	
Cumulative variance explained % (before rotation)	40.527	77.263	68.790	59.151	
Eigen root value (after rotation)	3.266	2.375	2.909	3.039	
Explanation of variance % (after rotation)	21.776	15.832	19.393	20.262	
Cumulative variance explained % (after rotation)	21.776	77.263	61.431	42.038	
KMO value	0.889				
Bartlett’s Test of Sphericity *χ*^2^	6265.063				
df	105				
*p*-value	0.000				

Factor loadings greater than 0.70 are shown in bold, indicating strong associations between items and their corresponding factors.

To verify the reliability as well as the validity of our model, we conducted a CFA to examine the factor structure and item loadings for all of the focal variables in our study. Cronbach’s *α* should be at least >0.5 and preferably >0.7 (Anderson and Gerbing, 1988). The Cronbach’s α values for the four factors in this study were all above 0.8, indicating good measurement reliability.

AMOS 24.0 software was adopted to calculate convergent and discriminant validity tests. The measurement model fitted well with *χ*^2^ (193.29)/df (84) = 2.301, GFI (goodness-of-fit index) = 0.962, CFI (comparative fit index) = 0.982, NFI (normed fit index) = 0.969, IFI(incremental fit index) = 0.982, TLI (Tucker Lewis index) = 0.978, RFI (relative fit index) = 0.962, PNFI (Parsimony-Adjusted) = 0.776, RMSEA (root mean square of error of approximation) = 0.041 with a 95% confidence interval of [0.033, 0.048]. As shown in Table 5, all the values of indices meet the satisfactory fit standard, indicating that the measurement model fit for this validation was good.

**Table 5:** Analysis of the degree of fit of the measurement model.

Indicator	CMIN/DF	GFI	CFI	RMSEA	NFI	RFI	IFI	TLI	PGFI	PNFI
Measured value	2.301	0.962	0.982	0.041	0.969	0.962	0.982	0.978	0.673	0.776
Acceptable fit standard	<5	>0.9	>0.9	<0.08	>0.9	>0.9	>0.9	>0.9	>0.5	>0.5
Satisfactory fit standard	<3	≥0.9	>0.9	<0.05	≥0.9	≥0.9	≥0.9	≥0.9	>0.5	>0.5
Conformity	YES	YES	YES	YES	YES	YES	YES	YES	YES	YES

As shown in Table 3, fit indices and AVE for the constructs of the measurement model, the standardized loadings of each indicator on the corresponding factor were significant (*p* < 0.001) and were above 0.70. Moreover, the AVE (average variance extracted) value for each variable was over 0.50, and the CR (composite reliability) of each variable was over 0.70, demonstrating good convergent validity of the scales.

As for the discriminant validity, the square root of AVE for each variable, as shown on the diagonal in Table 6, exceeded the correlation between latent variables, indicating adequate discriminant validity. Hence, the discriminant validity of the current structural model is satisfied (Fornell and Larcker, 1981). The HTMT was also used to assess discriminant validity (see Table 7). The results showed that all the values were lower than 0.9 (Anderson and Gerbing, 1988; Ali et al., 2018), suggesting discriminant validity within the data.

**Table 6:** Results of discriminant validity tests.

Discriminant validity analysis	AI Information encounter	Cognitive flexibility	AI literacy	Creativity
AI information encounter	0.858			
Cognitive flexibility	0.232	0.831		
AI literacy	0.162	0.529	0.811	
Creativity	0.442	0.553	0.510	0.837

**Table 7:** Results of HTMT.

Discriminant validity analysis	AI Information encounter	Cognitive flexibility	AI literacy	Creativity
AI information encounter				
Cognitive flexibility	0.233			
AI literacy	0.159	0.233		
Creativity	0.443	0.559	0.513	

Using AMOS 24.0, we ran a three-step multi-group CFA (configural, metric, and scalar) across gender, grade, and major domain (Meredith, 1993). Configural fit was acceptable; metric and scalar stages were evaluated with ΔCFI ≤ 0.010 and ΔRMSEA ≤ 0.015. Results indicate that our measures are broadly invariant across groups, with only minor, theory-guided partial-scalar adjustments, supporting strong overall cross-group comparability. See Appendix B for details of the measurement invariance tests.

#### 4.3.3 Main effect and mediation effect

To control for heterogeneity in responses due to contextual factors (Bernerth and Aguinis, 2016), gender and grade are often controlled in human information behavior (Stewart and Basic, 2014). Students’ major was also involved in the control variable, as different subject knowledge might correlate with the competence to collaborate with AI.

For all latent variables used in the PROCESS analysis, we computed overall scores as the grand mean of their respective domain scores. For example, the overall creativity score was calculated as the mean of four domain-level subscale scores: everyday, scholarly, scientific, and artistic creativity. These subscale scores were themselves derived by averaging the corresponding measurement items within each dimension (e.g., the “everyday” creativity score was computed as the mean of its three items). This scoring approach ensured that each domain contributed equally to the overall construct while preserving the multidimensional structure of the scale.

In examining the direct and indirect effects between AIIE, CF, and CRE, we employed Hayes’s PROCESS Model 4 with 5,000 bootstrap resamples and the default random sampling seed provided by the PROCESS macro (Hayes, 2018). All continuous variables were mean-centered prior to analysis. The bootstrap analysis revealed that AIIE significantly predicts CRE, with the total effect of AIIE on CRE registering *β* = 0.3178 [CI = (0.2653, 0.3703), *p*<0.0001]. As for the control variables, the effect of gender and class on creativity was reported as insignificant, while major was negatively correlated with creativity [*β* = −0.0776, CI = (−0.1117, –0.0435), *p*<0.0001]. As for the mediation test, the indirect effect of AIIE via CF on CRE was positive and significant [*β* = 0.0653, 95% bias-corrected, CI = (0.0402, 0.0928)]. Thus, H2 was supported. Notably, when CF joined the model, the direct effect of AIIE on CRE was still significant [*β* = 0.2525, CI = (0.2037, 0.3013), *p*<0.0001]. Thus, CF serves as a partial mediator in the path AIIE→CF→CRE.

#### 4.3.4 Moderated effect test

In examining the AIL as the moderator between AIIE and CF, and between AIIE and CRE, we conducted PROCESS Model 1 with 5,000 bootstraps (Hayes, 2018). The interaction term (AIIE×CRE) was constructed from mean-centered variables and is itself centered.

**Figure 2:**
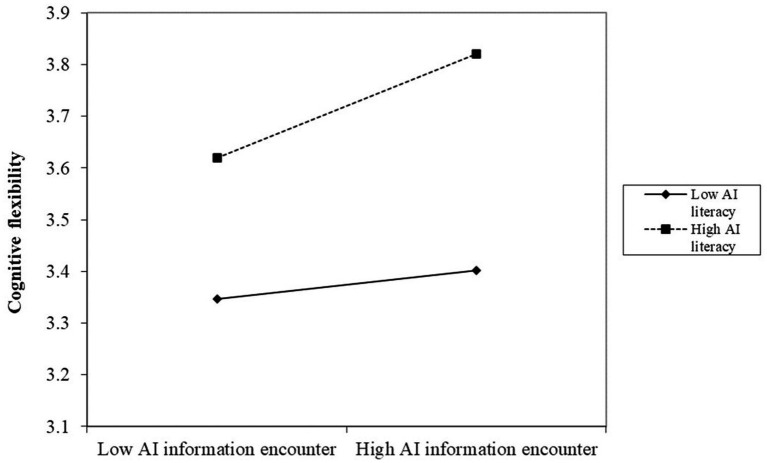
Association between AIIE and CF across AI literacy levels.

**Figure 3:**
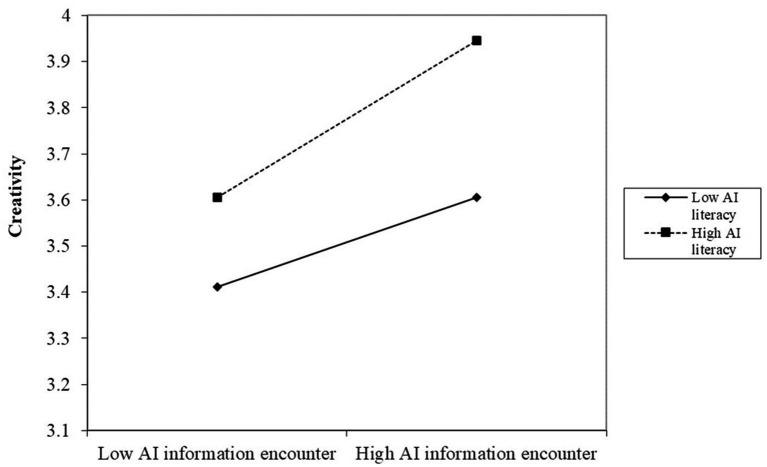
Association between AIIE and CRE across AI literacy levels.

The results show a significant positive interplay of AIIE×AIL on CRE path [*β* = 0.2120, *p*<0.001, CI = (0.0794, 0.3446)], as shown in model 3 in Table 8, supporting H5. To understand the relationship of AIL, AIIE, and CF, the effect of the interplay of AIIE×AIL on CF was also examined, which exhibits a positive effect as shown in model 2 in Table 8 [*β* = 0.2101, *p*<0.001, CI = (0.0529, 0.3672)]. The simple slope analysis (shown in Figures 2, 3) depicts that for students with higher (M + 1SD) AIL, AIIE was a stronger positive predictor of CF and CRE than for the students with lower (M − 1SD) AIL, supporting H3.

**Table 8:** The output of moderated mediation analysis.

Predictor	Model 1			Model 2			Model 3		
	CF			CRE			CRE		
	*β*	SE	*t*-value	*β*	SE	*t*-value	*β*	SE	*t*-value
Constant	4.1587**** [1.8743, 6.4430]	1.1633	3.5749	4.1865**** [2.2592, 6.1138]	0.9815	4.2656	3.1267**** [1.2697, 4.9837]	0.9457	3.3063
AIIE	0.6843* [−1.3006, –0.0679]	0.3139	−2.1799	−0.5476* [−1.0676, –0.0275]	0.2648	−2.0677	−0.3732 (ns) [−0.8712, 0.1248]	0.2536	−1.4716
AIL	−0.2811 (ns) [−0.8659, 0.3036]	0.2978	−0.9442	−0.4000 (ns) [−0.8933, 0.0933]	0.2512	−1.5921	−0.3283 (ns) [−0.7993, 0.1427]	0.2398	−1.3689
AIIE×AIL	0.2101 *** [0.0529, 0.3672]	0.0800	2.6252	0.2120*** [0.0794, 0.3446]	0.0675	3.1396	0.1584** [0.0313, 0.2856]	0.0648	2.4465
CF							0.2548**** [0.1923, 0.3174]	0.0319	7.9973
Gender	0.1088*** [0.0477, 0.1699]	0.0311	3.4942	0.0464(ns) [−0.0052, 0.0980]	0.0263	1.7675	0.0187 (ns) [−0.0310, 0.0684]	0.0253	0.7393
Grade	0.0537*** [0.0237, 0.0838]	0.0153	3.5084	0.0183(ns) [−0.0071, 0.0436]	0.0129	1.4124	0.0046 (ns) [−0.0199, 0.0290]	0.0124	0.3661
Major	−0.0248(ns) [−0.0568, 0.0072]	0.0163	−1.5244	−0.0853 (ns) [−0.1123, –0.0583]	0.0137	−6.2078	−0.0790*** [−0.1048, –0.0532]	0.0131	−6.0133
*R*	0.5224****, *F* (6,638) = 39.9195			0.6222****, *F* (6,638) = 67.1769		0.6656****, *F* (7,637) = 72.3988			
*R* ^2^	0.2729			0.3872		0.4431			

***p < 0.001, **p < 0.01, *p < 0.05. AIIE, AI information encounter; AIL, AI literacy; CF, cognitive flexibility; CRE, creativity.

**Figure 4:**
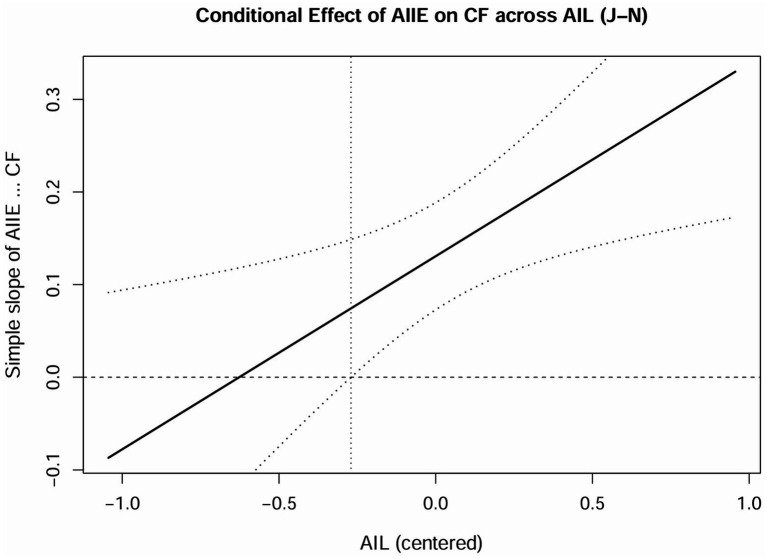
Conditional effect of AIIE on CF across AIL (J–N).

**Figure 5:**
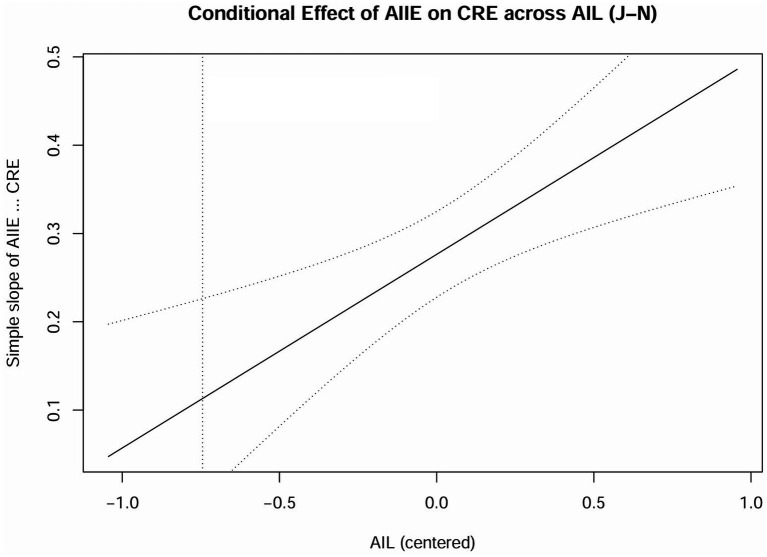
Conditional effect of AIIE on CRE across AIL (J–N).

We adopted the Johnson-Neyman technique to illuminate the entire range of a continuous moderator variable to highlight the areas of significant and insignificant moderated effect by the Johnson-Neyman point (Hayes, 2018; Spiller et al., 2013). For the moderation between the effect of AIIE and CF, the Johnson-Neyman point of AIL value was 3.6234, where AIIE significantly relates to CF within AIL at an interval of [2.8333, 3.6234], and insignificantly within AIL at an interval of [3.6234, 4.8333]. For AIIE’s effect on CRE, the Johnson-Neyman point of AIL value was 3.0696, where AIIE significantly has a positive association with CF within AIL at an interval of [2.8333, 3.0696], and insignificantly within AIL at an interval of [3.0696, 4.8333]. We used R to plot two Johnson–Neyman (J–N) figures: the conditional effect of AIIE on CF across AIL (see Figure 4) and on CRE across AIL (see Figure 5). The vertical line(s) divide the moderator range into regions where the simple slope of AIIE was significant versus non-significant (bands show 95% CIs).

#### 4.3.5 Moderated mediation effect test

We conducted the PROCESS Model 8 to examine the moderated-mediation models in line with our theoretical model (Hayes, 2018). AIIE (X) relates to creativity (Y) indirectly via cognitive flexibility (M), and AI literacy (W) moderates both (i) the AIIE→CF path [*β* = 0.2101, CI = (0.0529, 0.3672), *p<*0.001] and (ii) the direct AIIE→CRE association. When incorporating CF as a mediator (Table 8), the AIIE×AIL interaction on CRE remained significant [*β* = 0.1584, 95% CI (0.0313, 0.2856), *p* < 0.01], supporting H4. The direct effect of AIIE on CRE became non-significant once both moderation and mediation were included [*β* = −0.3732, 95% CI (−0.8712, 0.1248), *p* = 0.142].

Moreover, the index of moderated mediation was significant at 0.0535 [CI = (0.0068, 0.1038)]. In line with the specified model, the indirect association AIIE→CF→CRE varies with AIL (see Table 9), with the conditional indirect effects being probed for significance at + − 1 SD. The results revealed that the indirect effects of AIIE on CRE via CF were significantly stronger when in the higher AIL group (M + 1SD) rather than the lower AIL group (M − 1SD), with the contrast between these two conditional indirect effects significantly registering 0.0379 [CI = (0.0048, 0.0734)]. Notably, the mediation effect of CF diminished to insignificance when the students reported low AIL, further supporting H4 that the mediated effect is stronger at a high level of AI literacy than at a low level.

**Table 9:** Conditional indirect effect of AIIE on CRE via CF.

Path	AIL level	Indirect effect	95% confidence interval		
			BootSE	BootLLCI	BootULCI
AIIE→CF→CRE	3.5240 (M-1SD)	0.0143	0.0127	−0.0095	0.0398
	3.8776 (M)	0.0332	0.0093	0.0166	0.0533
	4.2313 (M + 1SD)	0.0522	0.0129	0.0289	0.0791
	Contrast	0.0379	0.0175	0.0048	0.0734

The partial *R*^2^ of AIIE predicting creativity was 0.146, indicating that AIIE explained about 14.6% of the residual variance in creativity after controlling for CF, AIL, and demographics. Conceptually, an unobserved confounder would need to account for a similarly large proportion of variance in both AIIE and creativity to nullify this effect, suggesting the relationship is substantively robust. As a cautionary note, PROCESS (Models 4 and 8) estimates indirect and conditional indirect associations under sequential ignorability. In cross-sectional data, these are best viewed as statistical decompositions rather than causal mechanisms.

#### 4.3.6 Supplementary model validation

To enhance the robustness and methodological rigor of our findings, we conducted a complementary analysis using Partial Least Squares Structural Equation Modeling (PLS-SEM). While the PROCESS macro in SPSS is effective for testing mediation and moderation effects in relatively simple path models, PLS-SEM provides a more comprehensive framework for validating complex structural models, including both measurement quality and the interplay among multiple latent constructs.

PLS-SEM was selected for several reasons. As a variance-based method, it requires fewer assumptions regarding residual distributions, multivariate normality, and measurement scale properties, making it particularly well-suited for bootstrap-based analysis in social science research (Chin et al., 2003; Hair et al., 2017). Moreover, given that AIIE is a newly developed construct adapted from the traditional IE framework, PLS-SEM is especially appropriate due to its strength in exploratory theoretical modeling and new scale validation (Hair et al., 2011; Ringle et al., 2012).

##### 4.3.6.1 Structural model

**Figure 6:**
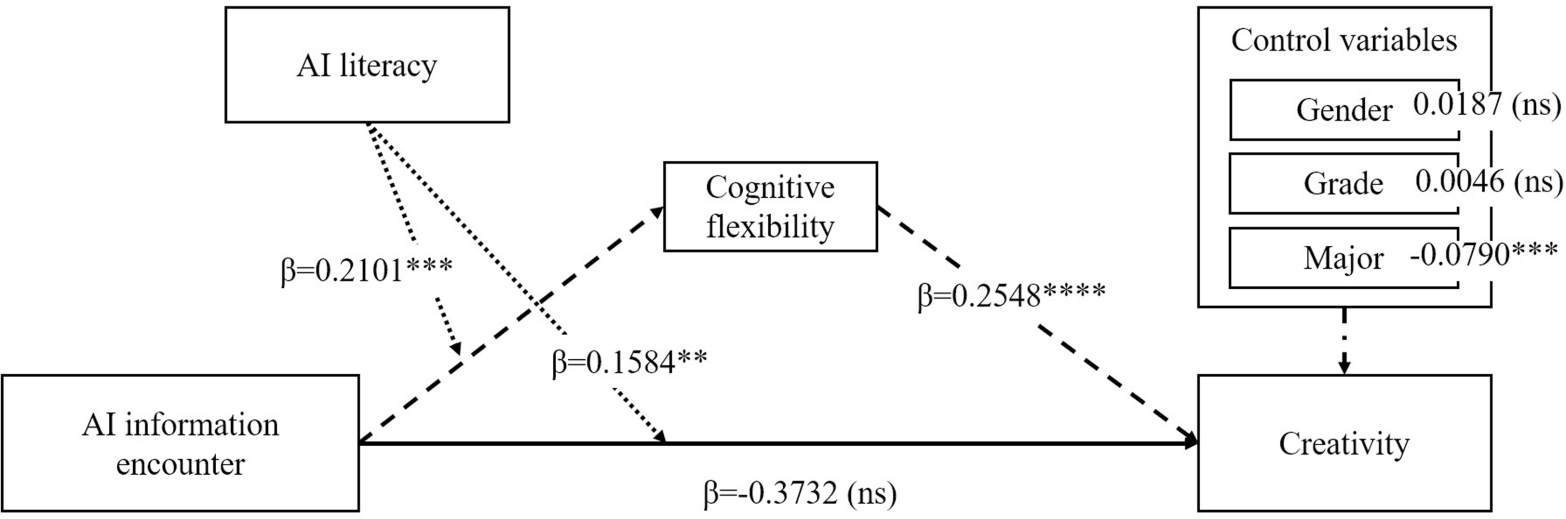
The results of moderated mediation analysis. Index of moderate mediation = 0.0535, 95% confidence interval CI = [0.0068, 0.1038]. *p<0.05, **p<0.001, ***p<0.0001.

To test the research model, bootstrapping with 1,000 subsamples was employed to analyze the relational assessments and the hypotheses using PLS-SEM (Hair et al., 2011). The structural model also exhibit good fitness with *χ*^2^ (387.764)/df (147) = 2.637, CFI (comparative fit index) = 0.954, NFI (normed fit index) = 0.939, IFI (incremental fit index) = 0.973, TLI (Tucker Lewis index) = 0.961, RFI (relative fit index) = 0.969, PNFI (Parsimony-Adjusted) = 0.801, RMSEA(root mean square of error of approximation) = 0.063 with a 95% confidence interval of [0.043, 0.062]. All the values of indices meet the satisfactory fit standard, indicating that the SEM fit for this validation was good. To estimate the accuracy of the structural framework, the *R*^2^ of variance explained for CF (*R*^2^ = 0.243, adjusted *R*^2^ = 0.239) and CRE (*R*^2^ = 0.405, adjusted *R*^2^ = 0.401) were calculated as predictive power (Hair et al., 2011). As shown in Figure 6, all eight hypotheses were supported.

Specifically, AIIE was found to significantly relate to CRE (H1: *β* = 0.336, *t* value = 11.126, *p* < 0.0001), with the direct effect of *β* = 0.295 (*t* value = 9.909, *p* < 0.0001). The indirect path of AIIE→CF→CRE was also positively significant (H2: *β* = 0.040, *t* value = 3.366, *p* = 0.001). For the moderating effect, the direct effect of AIIE×AIL on CRE was *β* = 0.105 (*t* value = 3.245, *p* = 0.001), supporting H3. Additionally, the direct effect of AIIE×AIL on CRE also yields significance (*β* = 0.084, *t* value = 2.029, *p* = 0.042). For the moderated mediation test, the indirect effect of AIIE×AIL→CF→CRE shows significance (H4: *β* = 0.025, *t* value = 1.979, *p* = 0.048), as shown in Table 10.

**Table 10:** Total, direct and indirect effects under PLS-SEM.

Path	Total effect			Direct effect			Indirect effect		
	*β*	*t*-value	*p*-value	*β*	*t*-value	*p*-value	*β*	*t*-value	*p*-value
AIIE→CRE	0.336	11.126	0.000	0.295	9.909	0.000			
AIIE→CF	0.136	3.847	0.000	0.136	3.847	0.000			
CF→CRE	0.296	8.353	0.000	0.296	8.353	0.000			
AIIE×AIL→CF	0.084	2.029	0.042	0.084	2.029	0.042			
AIIE×AIL→CRE	0.105	3.245	0.001	0.080	2.681	0.007			
AIIE→CF→CRE							0.040	3.366	0.001
AIIE×AIL→CF→CRE							0.025	1.979	0.048

AIIE, AI information encounter; AIL, AI literacy; CF, cognitive flexibility; CRE, Creativity.

##### 4.3.6.2 Inclusion of control variables

To assess the robustness of the research model and account for potential demographic influences, gender, major, and grade were included as control variables in a PLS-SEM analysis with 1,000 bootstrap resamples. To estimate the accuracy of the structural framework, the *R*^2^ of variance explained for CF (*R*^2^ = 0.243, adjusted *R*^2^ = 0.239) and CRE (*R*^2^ = 0.438, adjusted *R*^2^ = 0.431) were calculated, which is slightly higher than the model without control variables, showing better fitness of the present model. As shown in Table 11, all eight hypotheses were supported.

Specifically, AIIE was found to significantly relate to CRE (H1: *β* = 0.341, *t* value = 11.575, *p* < 0.0001), with the direct effect of *β* = 0.304 (*t* value = 10.350, *p* < 0.0001). The indirect path of AIIE→CF→CRE was also positively significant (H2: *β* = 0.120, *t* value = 6.384, *p* < 0.0001). For the moderating effect, the direct effect of AIIE×AIL on CRE was *β* = 0.093 (*t* value = 3.074 *p* = 0.002), supporting H3. Additionally, the direct effect of AIIE×AIL on CRE also yields significance (*β* = 0.070, *t* value = 2.520, *p* = 0.012). For the moderated mediation test, the indirect effect of AIIE×AIL→CF→CRE shows significant (H4: *β* = 0.023, *t* value = 1.960, *p* = 0.050).

In terms of controlled variables, major and gender showed a nonsignificant effect on creativity (major: *β* = 0.016, *t* = 0.523, *p* = 0.601; gender: *β* = 0.055, *t* = 0.834, *p* = 0.404). Notably, grade exhibited a significant negative association with creativity (*β* = −0.184, *t* = 5.843, *p* < 0.001), which may be attributed to senior students’ increasing familiarity with structured academic paradigms and the resulting cognitive rigidity as they progress through their studies, thereby reducing opportunities to foster creativity. When the impact of the control variables was considered, the results still confirmed that the model remained stable and significant. As shown in Table 11, the analytical data supported H1-5, thereby supporting its robustness against additional variable bias.

**Table 11:** Total, direct and indirect effects under PLS-SEM (with controlled variable).

Path	Total			direct			indirect		
	*β*	*t*-value	*p*-value	*β*	*t*-value	*p*-value	*β*	*t*-value	*p*-value
AIIE→CRE	0.341	11.575	0.000	0.304	10.350	0.000			
AIIE→CF	0.136	3.847	0.000	0.136	3.847	0.000			
CF→CRE	0.274	7.716	0.000	0.274	7.716	0.000			
AIIE×AIL→CF	0.084	2.029	0.042	0.084	2.029	0.042			
AIIE×AIL→CRE	0.093	3.074	0.002	0.070	2.520	0.012			
AIIE→CF→CRE							0.120	6.384	0.000
AIIE×AIL→CF→CRE							0.023	1.960	0.050
Major→CRE	0.016	0.523	0.601	0.016	0.523	0.601			
Grade→CRE	−0.184	5.843	0.000	−0.184	5.843	0.000			
Gender→CRE	0.055	0.834	0.404	0.055	0.834	0.404			

In addition, we conducted a covariance-based Structural Equation Modeling (SEM) analysis using AMOS 24.0 software (see Appendix C). The results of the structural model replicated the findings observed in our primary analyses, further supporting the validity of the hypothesized relationships. By incorporating multiple analytical approaches, including PROCESS modeling, PLS-SEM, and covariance-based SEM via AMOS (Hair et al., 2011), we cross-validated the core effects through distinct estimation frameworks commonly applied in survey-based research. This multi-method strategy enhances the robustness and generalizability of our findings.

## 5 Findings and discussion

Dewett and Gruys (2007) posit that innovation success fundamentally depends on creativity levels. Given university students’ pivotal role as creativity reservoirs, cultivating creativity within educational systems has become a strategic priority across nations. However, empirical evidence reveals persistent gaps in operationalizing creativity development through higher education pedagogies (Wu et al., 2014). Therefore, it is essential to examine the interaction mechanisms of internal and external factors in fostering student creativity in order to enable targeted interventions to optimize creativity cultivation frameworks. Drawing on cognitive theory, AIIE is linked to creativity through cognitive flexibility, while AI literacy filters and guides the evaluation and integration of useful information within AIIE.

A survey on sample of 645 university students across different genders, majors, and grades provides general support for our hypotheses. First, it was found that AIIE had a strong predictive effect on creativity (H1 was supported), which is consistent with the findings of previous studies on IE and innovation elicitation at the group level (Benedek et al., 2023; Palani et al., 2021; Busch, 2024). Notably, the results could only be interpreted in a way of association, but not a causal relationship.

Second, the mediation test indicated that AIIE was associated with higher CF, and CF accounted for an indirect association between AIIE and creativity. The results are in line with CFT: the multifaceted and hyper-text information, akin to the features of information content in AIIE, could reconstruct cognitive schema and develop more flexible knowledge representations in the face of different situations, thereby promoting creativity (Spiro, 1987; Spiro et al., 1988). Notably, CF plays a partial mediation role in this context, with the direct effect remaining significant. Though CF is legible in elucidating how AIIE triggers creativity, there are potential underlying explanation paths for the main effect.

Third, the findings revealed AI literacy as a moderating role in AIIE and validated the moderated mediation. Our study first regards AI literacy as a filter for capturing useful information for fostering individual creativity. Unlike previous studies that deemed information literacy, a concept akin to AI literacy, as a direct stimulus in human information behavior (Stewart and Basic, 2014), our results indicate that AI literacy is statistically related to the strength of the association between multifaceted, ill-structured information and cognitive flexibility. Notably, when students are at a lower level of AI literacy, the mediation effect of cognitive flexibility disappears. Namely, sole exposure to AIIE without good AI literacy only serves as a necessary but not sufficient condition for the CFT effect. Therefore, fostering students’ AI literacy as well as accumulating AIIE are both crucial for cultivating creativity.

Fourth, we also find that the control variable, grade, negatively predicts creativity. This may reflect a shift in upper-year students’ focus from exploratory learning to more outcome-driven academic and career objectives. In addition, cognitive fixation, domain specialization, and accumulated academic fatigue may reduce opportunities or motivation for creative thinking as students advance through their studies. This finding further supports our theoretical proposition that cognitive flexibility serves as a crucial pathway in the positive correlation between AIIE and creativity, particularly in overcoming the rigidity that may develop over time in academic trajectories.

Fifth, while the results align well with our proposed theoretical model and offer solid statistical support for the hypothesized relationships, we recognize that the cross-sectional survey design limits definitive causal interpretations. To enhance analytical rigor, we conducted additional robustness checks using both PLS-SEM and CB-SEM (Hair et al., 2011; Ringle et al., 2012), which consistently replicated the PROCESS findings and reinforced the predictive pathways from AIIE to CF and CRE. These converging results across different modeling approaches increase our confidence in the stability and generalizability of the model. Nonetheless, as all data were collected via self-report at a single time point, potential concerns about common method bias and shared-source variance remain. Future research may benefit from integrating instructor evaluations, behavioral creativity tasks, or longitudinal designs to further validate and extend these findings.

Finally, our results align with Tan and Maravilla’s (2024) argument that generative AI positively predicts academic integrity when embedded in designs that cultivate intrinsic motivation, digital literacy, and constructivist learning. First, the observed associations between AIIE and creativity suggest that structured AI-supported inquiry can promote students’ self-directed exploration, a motivational basis for authentic work rather than shortcut-seeking. Second, the behaviors we measure (e.g., noticing, checking, and capturing AI outputs) map onto critical digital literacy practices, evaluating sources, documenting provenance, and refining prompts, which reduce opportunities for misuse. Third, the iterative engagement we document is consistent with constructivist principles, where learners actively make meaning from AI-mediated encounters rather than passively reproducing outputs. AI literacy is not merely technical competence, but an ethical and reflective capacity that helps students navigate the tensions between convenience and integrity, and mitigates AI-induced cognitive dissonance in academic work (Seran et al., 2025). Framing our findings within this integrity-centered approach highlights how ethical implementation (transparent policies, scaffolded reflection, and assessment designs that reward process) can simultaneously discourage misconduct and promote genuine learning with generative AI (Tan and Maravilla, 2024).

## 6 Conclusion

### 6.1 Theoretical contribution

First, this study pioneered investigating the effect and mechanism of AIIE in fostering university student creativity, identifying a novel stimulus in influencing individual creativity in human-AI information interaction. In recent years, as individuals increasingly interact with user-friendly AI systems and apps, the likelihood of accidentally encountering unexpected information has grown, prompting research into the value of IE, or information serendipity in human information behavior (Liu et al., 2022). Moreover, previous studies mainly focused on the antecedents of IE but largely ignored the outcomes, especially individual creativity (Björneborn, 2017; Busch, 2024; Lievens et al., 2022; Kim et al., 2023; Cunha et al., 2015; Thakuria et al., 2024). The present study echoes this demand and complements the research of IE by advancing the research on information reuse and value realization after IE. Specifically, this study introduces the concept of AIIE in the context of human-AI interaction for the first time and empirically validates its effect and path on creativity. Therefore, our study not only expands the understanding of a novel context of IE and its unexplored outcome, creativity, but also provides new insights for specific AI-human information interaction in fostering individual creativity.

Second, by elucidating the mediating role of cognitive flexibility in AIIE, enhancing creativity, we enrich the understanding of how information behavior is positively associated with creativity from a novel theoretical lens. Existing research has primarily focused on the correlation between CF and individual lives (Diamond and Lee, 2011; Dajani and Uddin, 2015) or examined the driving factors regarding personal characteristics (Chung et al., 2012; Jacques and Zelazo, 2013). Although cognitive flexibility has been acknowledged as a key driver of creativity (Chen et al., 2019; Dietrich and Kanso, 2010; Hohl and Dolcos, 2024), studies addressing the antecedents of cognitive flexibility in terms of informational stimuli remain scarce. We draw understanding from CFT and explore the trigger of CF in AI-driven information encountering. In an ill-structured and multi-faceted information landscape, students reorganize cognitive schema into more dynamic and flexible representations. By revealing the “black box” between AIIE and individual creativity, we complement the underlying mechanism of IE and creativity, and advance understanding of CFT in human-AI interaction information behavior.

Third, we advance the boundary condition in establishing creativity through IE in the AI context. Although CFT posits that exposure to complex information sparks cognitive schema change, previous research has inadequately addressed the boundary conditions (Hu and Spiro, 2021; Jang, 2000). Particularly in human-AI interaction contexts involving serendipitous information acquisition, cognitive flexibility is not universally attainable across individuals. Previous studies on AI-driven creativity enhancement predominantly focus on technological attributes (Paesano, 2023; Benvenuti et al., 2023) while neglecting the moderating effects of individual characteristics and competence. This study therefore, examines AI literacy as a boundary condition for the association between AIIE, cognitive flexibility, and creativity, proposing that AI literacy helps students filter and capture valuable AI-generated insights amidst bewildering information. Our findings extend CFT’s applicability boundary conditions and enrich the AI-human interaction literature by identifying pivotal moderating mechanisms in cultivating creativity effectively through AI-mediated communication.

### 6.2 Practical implications

First, teachers can encourage students to pay more attention to serendipitous information when interacting with AI, so as to receive sufficient stimuli from the external environment. By actively embracing these seemingly unfamiliar or unusual pieces of information, students can attempt to generate novel and unconventional ideas. Educators can leverage AI-assisted pedagogy to facilitate multidimensional knowledge transfer, for instance, by designing interdisciplinary case studies and introducing AI-generated contradictory information in course assignments. This approach stimulates divergent thinking (an important component of creativity) through structured debates comparing various AI-proposed methodologies, thereby fostering student multi-perspective analysis and cognitive schema reconstruction.

Second, institutions and instructors can broaden students’ perspectives on phenomena through pedagogical strategies, thereby fostering cognitive flexibility. Our findings support an indirect pathway whereby AIIE relates to creativity via CF. Practically, this points to designing tasks that help students evaluate and recombine discrete knowledge units under varied, ill-structured conditions. Although more mature than K–12 populations, university students remain in developmental phases of intelligence, social skills, and values, continuing to shape their self-cognitive systems. For instance, teachers could design and assign knowledge unit tasks in multiple and dynamic conditions (such as counter-factual hypothesis analysis) for students, and let students freely explore appropriate perspectives with AI assistance. In this way, students could establish many alternative paths to get from one part of the overall knowledge base to any other part, hence flexibility in responding to highly diverse new cases could be enhanced.

Third, universities are suggested to prioritize developing students’ AI literacy to unlock gains in cognitive flexibility and creativity. Empirically, AIIE was associated with higher CF only at higher levels of AI literacy. Accordingly, teachers could encourage and assist students in proactively responding to the current technological transformation. Specifically, higher education institutions should integrate AI literacy into the general education curriculum by designing structured courses or modules that explicitly address its four core pillars: identification, application, evaluation, and ethics. For example, a general education course could teach students how to recognize different types of AI systems, use generative AI tools for information gathering and creative tasks, critically assess AI-generated outputs for accuracy and bias, and understand the ethical implications of AI reliance. These modules can be embedded across disciplines, not limited to computer science or engineering, ensuring that students from the humanities, business, and arts also acquire these competencies. In addition, universities can offer AI readiness training during freshman orientation or as micro-credentials to ensure early exposure.

An essential aspect of AI literacy is ethical AI literacy. Low ethical dimensions of AI literacy could weaken students’ sense of responsibility and initiative in tasks (Bankins and Formosa, 2023; Ardichvili, 2022), thereby reducing their tendency to change their cognitive structures and make efforts toward innovation. To improve ethical AI literacy, educational institutions should integrate AI ethics into the curriculum, addressing issues like algorithmic bias and overreliance on AI. Curriculum could also be designed to promote reflective thinking, encouraging students to critically evaluate AI-generated content for relevance, accuracy, and bias. Moreover, AI ethics is also suggested to be a core competency for students’ future careers, with educational institutions balancing ethical literacy and technical skills to foster moral responsibility and proactive innovation. Consistent with emerging perspectives, integrating reflective and constructivist strategies into AI literacy programs can reduce cognitive dissonance and over-reliance on generative AI. Encouraging students to critically evaluate AI outputs and to maintain dual-draft writing processes helps preserve originality and confidence (Seran et al., 2025).

Additionally, at the policy level, education administrators and national curriculum authorities should recognize the strategic value of incidental AI information behavior as a driver of higher-order cognitive outcomes. Policies may incentivize the integration of AI-supported learning platforms, support faculty development in AI-assisted pedagogy, and include creativity-oriented outcomes as key performance indicators in university evaluations. Importantly, given the reliance of creativity development on students’ access to and competence with AI technologies, equity in AI literacy education must also be prioritized. National or regional education authorities could consider mandating baseline AI literacy standards as a graduation requirement or accreditation criterion. Ministries of education are suggested to fund AI literacy curriculum development and support professional development programs to train faculty in integrating AI tools into teaching. Furthermore, targeted grants can be allocated to bridge digital equity gaps, ensuring that under-resourced institutions and disciplines have access to up-to-date AI tools and literacy support systems.

In sum, fostering creativity in university students in the AI era requires more than promoting tool use. It requires a systematic integration of AI literacy, flexible cognition, and serendipitous learning design across educational practice and policy.

### 6.3 Limitations and future research

There are several limitations in this study. Firstly, a limitation of this study is the gender imbalance in the sample, with a higher proportion of female participants (70.23%) compared to male participants (29.77%). This imbalance may be attributed to the higher participation rate of women in online surveys, as studies have indicated that women are often more willing to engage in online research (e.g., Salganik et al., 2006). To enhance generalizability, future research could aim for a more balanced gender representation, potentially by targeting a broader range of academic disciplines and exploring the factors influencing gender differences in online survey participation.

Second, this study adopts a quantitative, cross-sectional research design to examine the relationship between AI Information Encounter (AIIE) and creativity. While the statistical results provide support for the hypothesized model, the observed associations should be interpreted with caution, as the cross-sectional nature of the data limits the ability to draw firm conclusions about causality. It is possible that the positive link between AIIE and creativity develops over time and is shaped by dynamic, context-dependent factors. Although validated multi-item scales were employed, the mono-method nature of the data may limit the robustness of the observed effects. Future research could adopt longitudinal surveys or mixed-method approaches to explore temporal dynamics and capture the complex influence of AIIE.

Third, this study only explores the underlying mechanism from the cognitive flexibility theory perspective. Future researches are suggested to further explore other factors that mediate between IE and creativity, such as individual autonomy and value co-creation intention, to enrich more comprehensive motivational mechanisms of university student creativity.

Fourth, this study measured creativity through self-perceived abilities across four domains, which may not fully reflect behavioral or performance-based creativity. While subjectivity is a limitation, self-report measures remain valid for capturing students’ awareness of their creative behaviors, especially in educational contexts. Many items reflect past real-life behaviors, offering content validity. Future studies should incorporate behavioral creativity tasks (e.g., alternative uses, insight problems) and peer- or supervisor-rated outcomes to strengthen validity and more comprehensively examine the relationship between AI information encounter and creativity.

Fifth, despite controlling for gender, grade, and major, the analysis may still suffer from omitted-variable bias and endogeneity. Variables such as students’ AI use intensity, prior creativity experiences, cognitive or personality traits (e.g., openness, curiosity), and learning context were not included in this study, which might confound the relationships among AIIE, CF, AIL, and creativity. These unobserved covariates may bias the estimated associations and obscure causal interpretation. Future research should incorporate richer psychological and behavioral controls, employ longitudinal or experimental designs to strengthen causal inference, and mitigate endogeneity concerns.

Finally, our sample was obtained via judgmental sampling from a single Chinese online panel, which constrains external validity. Findings may not generalize to other populations or settings. Future studies should broaden recruitment across sites or regions and consider post-stratification weighting to enhance sample generalizability.

## Data Availability

The raw data supporting the conclusions of this article will be made available by the authors, without undue reservation.

## Footnotes

^1^ https://www.credamo.com
