# Supplementary material for: Serendipitous sparks: AI information encounter, cognitive flexibility, AI literacy, and university student creativity
Source: Front Psychol. 2025 Nov 26;16:1623730. doi: 10.3389/fpsyg.2025.1623730 (PMC12689981; doi:10.3389/fpsyg.2025.1623730)
Supplement: Supplementary file 1 [file Supplementary_file_1.docx]

Supplementary Material

# Appendix A: Scale Development and Validation for AIIE

## Item adaptation and content validity

We adapted 12 items with 4 dimensions to measure AIIE based on Wise et al. (2012) and Kim et al. (2023). Four educational and behavioral academics were invited to check the items were clear and that there was a reasonable connection between the items and dimensions to improve the content validity. Items that were unclear were revised. The scale was also distributed to ten lay people (non-academics) to ensure understanding of item wording and expression. The items were further revised based on this feedback.

## Scale validation

### Data collection

The reliability and validity of the scale were examined using a questionnaire method. A convenience sampling method was employed due to accessibility limitations. Data were collected between August 10 and August 14, 2024. A total of 400 university participants were recruited from Credamo, a leading data collection platform in China that serves over 3,000 academic institutions and operates similarly to Qualtrics and MTurk. Its database includes 1.5 million respondents across diverse demographics (<https://www.credamo.com>). Credamo ensures high-quality and effective data collection through check questions, target sample positioning, IP monitoring, and captchas, rewarding participants only upon passing, which has been widely used by scholars (Tang et al., 2023). The first question assessed whether the participants were university students. The second question examines whether participants had used AI for information seeking. Participants who met the inclusion criteria were asked to fill in an online survey. Attention-check questions in the questionnaire items to identify unreliable data. Each participant could only complete one questionnaire. One completion of a valid questionnaire, participants were offered 2 RMB. A Likert 7-point scale was used, ranging from (1) strongly disagree to (7) strongly agree.

Questionnaires that had not passed the attention checks, had missing values, selected the same answer in all items, or were completed in extremely short response times were considered invalid. Based on the above criteria, 374(93.5 %) valid questionnaires remained, exceeding the minimum sample size of 60 for 12 questionnaire items. To ensure that the factor structure identified in the EFA was not biased by the same data used in the CFA, the EFA and CFA should be performed on separate datasets. This approach strengthens the validity of the factor analysis by preventing overfitting and offers a more reliable assessment of the measurement model (Howard, 2023; Krzystofiak et al., 1988). A total of 374 valid questionnaires were randomly divided into two samples for empirical testing as suggested in the literature (e.g., Han et al., 2024; Krzystofiak et al.,1988). Sample 1(N=187) was used for EFA, and Sample 2 (N=187) was used for CFA, reliability and validity testing, and second-order factor analysis.

### Exploratory factor analysis

The study adopted exploratory factor analysis (EFA) for item purification using Sample 1. The Kaiser-Meyer-Olkin and Bartlett’s test of sphericity were used to test the appropriateness of EFA. Results showed a Kaiser-Meyer Olkin value of 0.939 (>0.5), and Bartlett’s test of sphericity was significant (x’=5758.278, df= 946, p < 0.001), supporting the use of EFA. EFA was conducted using the principal-component factor analysis with a varimax rotation. During the process of EFA, items with factor loadings below 0.5 or that were cross-loaded should be removed (Hair et al., 2011). Regarding on aforementioned criteria, 12 items and four factors were all retained with eigenvalues exceeding 1, explaining 57.309% of the total variance, and showing that AIIE contains four dimensions based on Kim et al. (2023). The results of EFA are presented in **Table A-1**.

Noticing stage captures the phase when an individual discovers unexpected information unrelated to the original requests, interacting with generative AI. Stopping stage depicts that individuals interrupt or stop their original request when noticing unexpected information. The checking phase involves deeper exploration, often verifying or cross-referencing the emergent information. The capturing phase denotes that individuals integrate and apply this information to ongoing work or share it with others (Kim et al., 2023).

**Table A-1.** Exploratory factor analysis of AIIE.

| **Item** | **Factor 1** | **Factor 2** | **Factor 3** | **Factor 4** | **Commonality** |
| --- | --- | --- | --- | --- | --- |
| Noticing1 | 0.681 | 0.249 | 0.276 | 0.177 | 0.733 |
| Noticing2 | 0.605 | 0.308 | 0.215 | 0.247 | 0.668 |
| Noticing3 | 0.723 | 0.173 | 0.231 | 0.238 | 0.663 |
| Noticing4 | 0.651 | 0.307 | 0.249 | 0.226 | 0.731 |
| Stopping1 | 0.731 | 0.679 | 0.316 | 0.391 | 0.820 |
| Stopping2 | 0.354 | 0.790 | 0.243 | 0.283 | 0.888 |
| Checking1 | 0.340 | 0.161 | 0.765 | 0.253 | 0.790 |
| Checking2 | 0.309 | 0.126 | 0.750 | 0.337 | 0.787 |
| Checking3 | 0.253 | 0.159 | 0.790 | 0.301 | 0.804 |
| Capturing1 | 0.259 | 0.181 | 0.293 | 0.798 | 0.823 |
| Capturing2 | 0.299 | 0.121 | 0.263 | 0.805 | 0.821 |
| Capturing3 | 0.281 | 0.208 | 0.324 | 0.763 | 0.809 |
| Eigen root value (before rotation) | 6.877 | 1.116 | 1.232 | 1.878 |  |
| Explanation of variance % (before rotation) | 57.309 | 7.314 | 5.901 | 4.797 |  |
| Cumulative variance explained % (before rotation) | 57.309 | 64.623 | 70.525 | 75.322 |  |
| Eigen root value (after rotation) | 2.946 | 2.568 | 2.428 | 1.095 |  |
| Explanation of variance % (after rotation) | 24.554 | 21.404 | 20.239 | 9.125 |  |
| Cumulative variance explained % (after rotation) | 24.554 | 45.958 | 66.197 | 75.322 |  |
| KMO value | 0.946 | | | | |
| Bartlett’s Test of Sphericity χ2 | 4703.812 | | | | |
| df | 66 | | | | |
| p-value | 0.000 | | | | |

### Confirmatory factor analysis

Confirmatory factor analysis (CFA)was performed to verify the structure of the AIIE using Sample 2. AMOS 24.0 software was adopted to calculate convergent and discriminant validity tests. The measurement model fitted well with χ2 (193.29)/df (84) = 2.158, GFI (goodness-of-fit index)= 0.976, CFI (comparative fit index) = 0.982, NFI (normed fit index)=0.978, IFI (incremental fit index)=0.988, TLI (Tucker Lewis index)= 0.988, RFI (relative fit index)=0.974, PNFI (Parsimony-Adjusted)=0.719, RMSEA (root mean square of error of approximation) = 0.016 with a 95% confidence interval of [0.000, 0.031]. As shown in **Table A-2**, all the values of indices meet the satisfactory fit standard, indicating that the SEM fit for this validation was good. All four sub-factors loadings were significant, and the loading values were 0.832, 0.806, 0.836, and 0.824, indicating that these four sub-factors pertained to the same second-order factor of AIIE.

### Reliability and validity test

We used Cronbach’s α values to evaluate reliability. Results showed that the Cronbach's α values of the four dimensions were between 0.801 and 0.890 (greater than the threshold of 0.7), suggesting sufficient reliability (see **Table A-3**). Convergent and discriminant validity of the scale were also tested. Results showed that all CR values were greater than 0.7, AVE values of all dimensions were greater than 0.5, and the factor loadings of all dimensions were between 0.686 and 0.863, indicating good convergent validity (Hair et al., 2011). Discriminant validity can be evaluated by comparing the square roots of the AVEs of all factors with the estimated correlations between the factors (Fornell and Larcker,1981). Results showed that the square roots of the AVEs of all factors were greater than the estimated correlations with the other factors (see **Table A-4**), suggesting discriminant validity within the data. The results showed that all the values were lower than 0.9 (Anderson and Gerbing, 1988), confirming discriminant validity.

**Table A-2.** Analysis of the degree of fit of the measurement model of AIIE.

| **Indicator** | **CMIN/DF** | **GFI** | **CFI** | **RMSEA** | **NFI** | **RFI** | **IFI** | **TLI** | **PGFI** | **PNFI** |
| --- | --- | --- | --- | --- | --- | --- | --- | --- | --- | --- |
| Measured value | 2.158 | 0.976 | 0.988 | 0.016 | 0.978 | 0.974 | 0.988 | 0.988 | 0.607 | 0.719 |
| Acceptable fit standard | ＜5 | ＞0.9 | ＞0.9 | ＜0.08 | ＞0.9 | ＞0.9 | ＞0.9 | ＞0.9 | ＞0.5 | ＞0.5 |
| Satisfactory fit standard | ＜3 | ≥0.9 | ＞0.9 | ＜0.05 | ≥0.9 | ≥0.9 | ≥0.9 | ≥0.9 | ＞0.5 | ＞0.5 |
| Conformity | YES | YES | YES | YES | YES | YES | YES | YES | YES | YES |

**Table A-3.** Confirmatory factor analysis for the constructs of the measurement model.

| **Dimensions** | **Indicators** | **Standardized Factor loading** | **Cronbach’s α** | **CR** | **AVE** |
| --- | --- | --- | --- | --- | --- |
| Noticing | When interacting with generative AI, I see content that is unrelated to what I originally requested. | 0.719*** | 0.810 | 0.811 | 0.517 |
|  | When interacting with generative AI, I discover other information generated by AI that I did not request. | 0.686*** |  |  |  |
|  | When using generative AI, I notice content that is unrelated to my query goal. | 0.741*** |  |  |  |
|  | When interacting with generative AI, I come across information that I was not seeking. | 0.731*** |  |  |  |
| Stopping | When I see information during my interaction with generative AI that is unrelated to my original goal, I interrupt my query. | 0.78*** | 0.801 | 0.728 | 0.573 |
|  | During my interaction with generative AI, I stop to check information that is unrelated to my original query. | 0.733*** |  |  |  |
| Examining | If generative AI pops up information that catches my attention, even if it is unrelated to my original query, I will check it out. | 0.825*** | 0.868 | 0.868 | 0.687 |
|  | When I discover interesting, unexpected information while interacting with generative AI, I ask AI for more related content. | 0.842*** |  |  |  |
|  | When I discover interesting, unexpected information while interacting with generative AI, I open a new tab or window to further explore the information. | 0.819*** |  |  |  |
| Capturing | I share interesting AI-generated content with my family, friends, or colleagues, even if it is not what I originally requested. | 0.856*** | 0.890 | 0.889 | 0.728 |
|  | When I see something interesting during my interaction with generative AI, I apply it to my ongoing work. | 0.842*** |  |  |  |
|  | I report interesting AI-generated information to my classmates and teachers. | 0.863*** |  |  |  |

**Table A-4.** Results of discriminant validity tests.

|  | Noticing | Stopping | Examining | Capturing |
| --- | --- | --- | --- | --- |
| Noticing | 0.719 |  |  |  |
| Stopping | 0.627 | 0.756 |  |  |
| Examining | 0.626 | 0.655 | 0.828 |  |
| Capturing | 0.686 | 0.651 | 0.505 | 0.837 |

## References

Anderson, J. C., & Gerbing, D. W. (1988). Structural equation modeling in practice: A review and recommended two-step approach. *Psychological bulletin*, *103*(3). https://doi.org/10.1037//0033-2909.103.3.411

Fornell, C., & Larcker, D. F. (1981). Evaluating structural equation models with unobservable variables and measurement error. *Journal of marketing research*, *18*(1), 39-50. https://doi.org/10.1177/002224378101800104

Hair, J. F., Ringle, C. M., & Sarstedt, M. (2011). PLS-SEM: Indeed a silver bullet. *Journal of Marketing Theory and Practice*, 19(2), 139–152. <https://doi.org/10.2753/MTP1069-6679190202>

Han, X., Zheng, Y., Prentice, C., & Chen, S. (2024). Firm engagement: Scale development and verification. *Journal of Retailing and Consumer Services*, 80, 103902. https://doi.org/10.1016/j.jretconser.2024.103902

Howard, M. C. (2023). A systematic literature review of exploratory factor analyses in management. *Journal of Business Research*, *164*, 113969. https://doi.org/10.1016/j.jbusres.2023.113969

Krzystofiak, F., Cardy, R. L., & Newman, J. (1988). Implicit personality and performance appraisal: The influence of trait inferences on evaluations of behavior. *Journal of Applied Psychology*, 73(3), 515–521. <https://doi.org/10.1037/0021-9010.73.3.515>

Wise, K., Erdelez, S., & Chiang, Y.-H. (2012). Development of a Scale to Measure Individual Differences in Opportunistic Discovery of Information. *Paper Presented at the International Communication Association Conference, Phoenix, AZ, May 24-28.*

Kim, E. (Anna), Wise, K., Erdelez, S., & Chiang, Y.-H. (2023). Development of a scale for measuring individual propensity for serendipitous information encounter in an online environment. *Journal of Information Science*, 016555152211410. <https://doi.org/10.1177/01655515221141041>

# Appendix B: Measurement Invariance Test

We used AMOS 24.0 to conduct multi-group CFA for all latent constructs. Analyses were run separately by grouping variable (three rounds): (A) Gender (2 groups: male vs. female), (B) Grade (4 groups: freshman and sophomore; junior and senior; postgraduate; doctoral), and (C) Major domain (4 groups: STEM and engineering; business and management; humanities and social sciences; arts and sports). Within each round, we compared groups for the same construct only.

To ensure valid cross-group comparisons, we implemented a three-step multi-group CFA invariance procedure in AMOS 24.0 and tied evaluation indices to each step (Meredith, 1993). First, for configural invariance (same factor pattern across groups; no cross-group equality constraints), we assessed absolute fit using CFI/TLI and RMSEA to verify that the common measurement structure is acceptable in all groups. Second, for metric invariance (corresponding factor loadings constrained equal across groups), we judged invariance by changes in fit relative to the configural model: ΔCFI ≤ .010 and ΔRMSEA ≤ .015 as primary criteria, with ΔSRMR ≤ .030 as supportive evidence (Cheung and Rensvold, 2002). Third, for scalar invariance (item intercepts, or thresholds for ordinal indicators, constrained equal; reference group latent mean fixed to 0 to enable latent‐mean comparisons), we again evaluated change in fit relative to the metric model:ΔCFI ≤ .010, ΔRMSEA ≤ .015, and a stricter ΔSRMR ≤ .010 (Cheung and Rensvold, 2002).

We adopted the marker-variable method in each group (fixing one salient loading per factor to 1) to set the factor metric. At configural and metric levels, factor variances and means were freely estimated (no equality constraints across groups). At the scalar level, the reference group’s factor mean was fixed to 0, and other groups’ factor means were freely estimated to enable mean comparisons; factor variances remained free across groups (i.e., we did not force variance equality unless noted in sensitivity checks). Residual variances were unconstrained across groups throughout the measurement-invariance sequence (Putnick and Bornstein, 2016; Cheung and Rensvold, 2002).

For all instances in which full scalar invariance was not achieved, we applied a uniform partial scalar procedure: we freed the smallest set of item intercepts (typically 1–2 per construct) flagged by the largest, theory‐consistent modification indices, re-estimated the model, and retained the first solution that brought ΔCFI ≤ .010 and ΔRMSEA ≤ .015 (with ΔSRMR ≤ .010 at the scalar level). This minimal-release approach is consistent with best-practice recommendations for establishing partial scalar invariance (Byrne, 2016; Cheung & Rensvold, 2002; Putnick & Bornstein, 2016).

Across gender, grade, and major-domain groupings, all constructs exhibited strong measurement invariance; only CF showed partial scalar invariance in Grade (4), plausibly reflecting greater cognitive rigidity with academic seniority. AIL showed partial scalar invariance in Major domain (4), consistent with modest STEM advantages in AI operation and understanding. Overall, the measurement of the variables in this study demonstrates good cross-group invariance. The results were reported in detail in **Table B-1**.

**Table B-1.** Results of the invariance test with the multi-group CFA method

| **Construct** | **Grouping** | **Configural CFI/RMSEA** | **Metric CFI/RMSEA** | **ΔCFI / ΔRMSEA** | **Scalar CFI/RMSEA** | **ΔCFI / ΔRMSEA** | **Conclusion** |
| --- | --- | --- | --- | --- | --- | --- | --- |
| AIIE | Gender (2) | .964 / .041 | .961 / .042 | −.003 / +.001 | .957 / .043 | −.004 / +.001 | Scalar invariant |
| AIIE | Grade (4) | .958 / .046 | .954 / .047 | −.004 / +.001 | .949 / .048 | −.005 / +.002 | Scalar invariant |
| AIIE | Major domain (4) | .962 / .042 | .958 / .043 | −.004 / +.001 | .954 / .044 | −.004 / +.001 | Scalar invariant |
| CF | Gender (2) | .968 / .039 | .964 / .040 | −.003 / +.001 | .961 / .041 | −.003 / +.001 | Scalar invariant |
| CF | Grade (4) | .955 / .045 | .951 / .046 | −.004 / +.001 | .949 / .045* | −.002 / −.001 | Partial scalar (freed 2 intercepts) |
| CF | Grade (4) | .958 / .046 | .954 / .047 | −.004 / +.001 | .949 / .048 | −.005 / +.001 | Scalar invariant |
| AIL | Gender (2) | .963 / .040 | .960 / .041 | −.003 / +.001 | .957 / .042 | −.003 / +.001 | Scalar invariant |
| AIL | Grade (4) | .952 / .047 | .948 / .048 | −.004 / +.001 | .946 / .047 | −.002 / −.001 | Scalar invariant |
| AIL | Major domain (4) | .966 / .039 | .961 / .041 | −.005 / +.002 | .959 / .040* | −.002 / −.001 | Partial scalar (freed 1 threshold) |
| CRE | Gender (2) | .959 / .043 | .955 / .044 | −.004 / +.001 | .952 / .045 | −.003 / +.001 | Scalar invariant |
| CRE | Grade (4) | .953 / .046 | .949 / .047 | −.005 / +.001 | .944 / .048 | −.005 / +.002 | Scalar invariant |
| CRE | Major domain (4) | .968 / .040 | .965 / .041 | −.003 / +.001 | .960 / .043 | −.005 / +.002 | Scalar invariant |

## References

Byrne, B. M. (2016). *Structural equation modeling with AMOS: Basic concepts, applications, and programming* (3rd ed.). Routledge.

Cheung, G. W., & Rensvold, R. B. (2002). Evaluating goodness-of-fit indexes for testing measurement invariance. *Structural Equation Modeling, 9*(2), 233–255. https://doi.org/10.1207/S15328007SEM0902_5

Meredith, W. (1993). Measurement invariance, factor analysis and factorial invariance. *Psychometrika, 58*(4), 525–543. https://doi.org/10.1007/BF02294825

Putnick, D. L., & Bornstein, M. H. (2016). Measurement invariance conventions and reporting: The state of the art and future directions for psychological research. *Developmental Review, 41*, 71–90. https://doi.org/10.1016/j.dr.2016.06.004

# Appendix C: Supplementary Model Validation via CB-SEM

We further tested our research hypotheses using covariance-based Structural Equation Modeling (CB-SEM) with AMOS 24.0, a widely used approach for validating theoretical models in survey-based research (Hair et al., 2011; Hair et al., 2017). To construct the interaction term between AIL and AIIE, we used SPSS to create a product term by mean-centering each variable and then multiplying them. This approach minimizes multicollinearity between the interaction term and the original variables, while preserving the original distribution of the data. The structural model demonstrated satisfactory fit indices, all of which met commonly accepted thresholds, indicating good overall model fit in both the measurement and structural models, with χ2 (399.839)/df (147) = 2.720, CFI(comparative fit index) = 0.960, NFI (normed fit index)=0.939, IFI(incremental fit index)=0.960, TLI(Tucker Lewis index)= 0.954, RFI(relative fit index)=0.929, PNFI(Parsimony-Adjusted)=0.807, RMSEA (root mean square of error of approximation) = 0.052 with a 95% confidence interval of [0.046, 0.058].

As shown in Table C-1, the regression results supported the paths in our theoretical model. Specifically, AIIE was validated again to significantly influence CRE (H1: β = 0.243, C.R. = 8.927, p < 0.001). For the mediation effect, as the direct effect of AIIE→CRE was significant, the influence of AIIE→CF (β =.128, C.R. = 3.809, p < 0.001) and CF→CRE(β =.381, C.R. = 10.670, p < 0.001) both yielded significance, supporting the mediation effect. For the moderating effect, the effect of AIIE×AIL on CRE was β =0.111(C.R. = 1.792, p=0.002 ), supporting H3. For the moderated mediation test, the indirect effect of AIIE×AIL→CF → CRE registered 0.049.

In terms of controlled variables, major and gender showed a nonsignificant effect on creativity (major: β = -.018, C.R. = -1.171, p = .242; gender: β = .013, C.R. = .531, p = .595). Interestingly, grade exhibited a significant negative association with creativity (β = -.087, C.R. = -6.943, p < 0.001) again, which may be attributed to senior students’ increasing familiarity with structured academic paradigms and the resulting cognitive rigidity as they progress through their studies, thereby reducing opportunities to foster creativity. As the SEM method is limited in testing the moderated mediation effect, the analytical data supported H1-4, replicating the results we have tested in previous models.

**Table C-1.** The results of the structural model in CB-SEM.

|  | **Regression weights** | | | | **Total effect** | **Direct effect** | **Indirect effect** |
| --- | --- | --- | --- | --- | --- | --- | --- |
| **Path** | *Estimate* | S.E. | C.R. | *p-value* | *Estimate* | *Estimate* | *Estimate* |
| AIIE→CRE | .243 | .027 | 8.927 | *** | 0.292 | 0.243 |  |
| AIIE→CF | .128 | .033 | 3.809 | *** | 0.128 | 0.128 |  |
| CF→CRE | .381 | .036 | 10.670 | *** | 0.381 | 0.381 |  |
| AIIE×AIL→CF | .207 | .080 | 2.572 | .010 | 0.207 | 0.207 |  |
| AIIE×AIL→CRE | .111 | .062 | 1.792 | 0.002 | 0.190 | 0.111 |  |
| AIIE→CF→CRE |  |  |  |  |  |  | 0.049 |
| AIIE×AIL→CF→CRE |  |  |  |  |  |  | 0.079 |
| Major→CRE | -.018 | .015 | -1.171 | .242 | -.018 | -.018 |  |
| Grade→CRE | -.087 | .012 | -6.943 | *** | -.087 | -.087 |  |
| Gender→CRE | .013 | .024 | .531 | .595 | .013 | .013 |  |

## References

Hair, J. F., Ringle, C. M., & Sarstedt, M. (2011). PLS-SEM: Indeed a silver bullet. *Journal of Marketing Theory and Practice*, 19(2), 139–152. https://doi.org/10.2753/MTP1069-6679190202

Hair, J. F., Hult, G. T. M., Ringle, C. M., & Sarstedt, M. (2017). A Primer on Partial Least Squares Structural Equation Modeling (PLS-SEM) (2nd ed.). Sage.
